# Obstructive and restrictive spirometry from school age to adulthood: three birth cohort studies

**DOI:** 10.1016/j.eclinm.2023.102355

**Published:** 2023-12-12

**Authors:** Anhar Ullah, Raquel Granell, Sadia Haider, Lesley Lowe, Sara Fontanella, Hasan Arshad, Clare S. Murray, Steve Turner, John W. Holloway, Angela Simpson, Graham Roberts, Adnan Custovic, STELAR/UNICORN Investigators, John Ainsworth, Philip Couch, Paul Cullinan, Graham Devereux, Ashley Woodcock

**Affiliations:** a National Heart and Lung Institute, Imperial College London, UK; b NIHR Imperial Biomedical Research Centre, London, UK; c Department of Population Health Sciences, Bristol Medical School, University of Bristol, UK; d Faculty of Biology, Medicine and Health, Division of Infection, Immunity and Respiratory Medicine, School of Biological Sciences, University of Manchester, Manchester Academic Health Science Centre, UK; e Faculty of Medicine, Human Development and Health, University of Southampton, Southampton, UK; f NIHR Southampton Biomedical Research Centre, University Hospitals Southampton NHS Foundation Trust, Southampton, UK; g David Hide Asthma and Allergy Research Centre, Isle of Wight, UK; h Royal Aberdeen Children's Hospital NHS Grampian Aberdeen, AB25 2ZG, UK; i Child Health, University of Aberdeen, Aberdeen, UK

**Keywords:** Lung function, Obstruction, Restriction, Worsening, Improvement

## Abstract

**Background:**

Spirometric obstruction and restriction are two patterns of impaired lung function which are predictive of poor health. We investigated the development of these phenotypes and their transitions through childhood to early adulthood.

**Methods:**

In this study, we analysed pooled data from three UK population−based birth cohorts established between 1989 and 1995. We applied descriptive statistics, regression modelling and data-driven modelling to data from three population−based birth cohorts with at least three spirometry measures from childhood to adulthood (mid-school: 8–10 years, n = 8404; adolescence: 15–18, n = 5764; and early adulthood: 20–26, n = 4680). Participants were assigned to normal, restrictive, and obstructive spirometry based on adjusted regression residuals. We considered two transitions: from 8–10 to 15–18 and from 15–18 to 20–26 years.

**Findings:**

Obstructive phenotype was observed in ∼10%, and restrictive in ∼9%. A substantial proportion of children with impaired lung function in school age (between one third in obstructive and a half in restricted phenotype) improved and achieved normal and stable lung function to early adulthood. Of those with normal lung function in school-age, <5% declined to adulthood. Underweight restrictive and obese obstructive participants were less likely to transit to normal. Maternal smoking during pregnancy and current asthma diagnosis increased the risk of persistent obstruction and worsening. Significant associate of worsening in restrictive phenotypes was lower BMI at the first lung function assessment. Data-driven methodologies identified similar risk factors for obstructive and restrictive clusters.

**Interpretation:**

The worsening and improvement in obstructive and restrictive spirometry were observed at all ages. Maintaining optimal weight during childhood and reducing maternal smoking during pregnancy may reduce spirometry obstruction and restriction and improve lung function.

**Funding:**

10.13039/501100000265MRC Grant MR/S025340/1.

**Research in context**

**Evidence before this study**

Two common phenotypes of impaired lung function in early adulthood which are predictive of subsequent poor long-term health outcomes are spirometric obstruction and restriction. Lung function trajectories through childhood are strong predictors of impaired spirometry in early adulthood. To date, no studies have investigated longitudinal development and transitions between obstructive and restrictive spirometry phenotypes (including deterioration and improvement) through childhood.

**Added value of this study**

The worsening and improvement in both phenotypes were observed at two transition points (8–10 to 15–18 years and 15–18 to 20–26 years). A substantial proportion of children with obstruction and restriction moved to normal phenotype (up to 50%), and some of those with normal lung function (∼5%) developing impairment. Importantly, the improvement was stable across the 2 time points. Underweight restrictive and obese obstructive participants were less likely to transition to normal. Significant associate of worsening in restrictive phenotypes was lower BMI at the first lung function assessment. Among current wheezers, the highest probability of persistent obstruction was for those with low gestational age, whereas the highest improvement was observed among those with higher gestational age.

**Implications of all the available evidence**

Within-individual changes in lung function over time (including improvement and worsening) do occur during childhood, and a substantial proportion of children with lung function impairment in early school age (between one third in obstructive phenotype and a half in restricted phenotype) improve and achieve normal and stable lung function to early adulthood. Efforts to reduce foetal growth restriction and premature birth, maintain optimal weight during childhood, and control maternal smoking during pregnancy may significantly reduce worsening in lung function.

## Introduction

Impairments in spirometric measures of lung function at the physiological peak in the third decade of life are associated with adverse health outcomes through life-course,^1^ including poor respiratory health and higher risk of COPD,^2^ but also cardiovascular and cerebrovascular events into middle age,^3^ sudden cardiac death^4^ and premature death of all causes.^5^ Overall evidence to date suggests that impaired spirometry in adulthood is associated with subnormal lung function trajectories through childhood.^6^ Therefore, understanding the factors associated with decline and improvement in lung function through childhood is important and may facilitate development of interventions to preserve or improve lung function and prevent subsequent onset and progression of ill health.

Two common phenotypes of diminished lung function which are predictive of different poor long-term health outcomes are spirometric obstruction and restriction; the obstructive phenotype has a reduced FEV_1_/FVC ratio, while the restrictive phenotype is characterized by a reduced FVC with a preserved FEV_1_/FVC. The prevalence of spirometry obstruction and restriction and their association with early risk factors have been studied in several adult cohorts, with substantial variation in findings.^7^ Two studies have reported cross-sectional data on prevalence, characteristics, and risk factors of these spirometry phenotypes during childhood.^8^^,^^9^ The prevalence of obstructive and restrictive spirometry during early childhood and young adulthood ranged from 3 to 11% and 2 to 8%, respectively.^9^ Known factors associated with spirometry obstruction are preterm birth, maternal smoking during pregnancy, family history of asthma and current smoking/environmental tobacco smoke (ETS) exposure.^8^, ^9^, ^10^ In contrast, factors associated with spirometry restriction are low body mass index (BMI) and other growth-related risk factors.^8^^,^^9^

Some previous studies have described “catch-up” and “growth failure” in specific spirometric measures of lung function,^11^ providing evidence that within-individual changes over time (such as improvement and worsening) do occur.^6^ However, to date, no studies have investigated longitudinal development and transitions between obstructive and restrictive spirometry phenotypes, including worsening and improvement. Filling this knowledge gap could provide insights into the early origins and transition behavior of these lung function phenotypes from childhood into adulthood and identify actionable targets to promote improvement and reduce worsening. To this end, we investigated the development of obstructive and restrictive lung function phenotypes and their transitions from school age to adulthood using different temporal frameworks and methodologies, from descriptive statistics and regression modelling to data-driven modelling. Firstly, we estimated the age and risk-specific prevalence of spirometric obstruction and restriction. We then explored the overall and age-specific transitions and the risk factors associated with transitions; finally, we used data-driven analyses to derive clusters of spirometry phenotypes over time.

## Methods

Detailed description of methods is presented in the Supplementary Appendix.

### Study design, setting, participants and data sources/measurements

We used data from three UK population−based birth cohorts with at least three measures of lung function from childhood to early adulthood: Manchester Asthma and Allergy Study (MAAS),^12^ the Avon Longitudinal Study of Parents and Children (ALSPAC),^13^ and Isle of Wight (IOW) cohort.^14^ Data were integrated to facilitate joint analyses.^15^ Details of clinical follow up and definitions of risk factors and clinical outcomes (including asthma and wheeze phenotypes from birth to early adulthood^16^) are provided in the Supplementary Appendix.

### Ethics statement

Research ethics committees approved all studies. Informed consent was obtained from parents, and participants gave their assent/consent when applicable.

### Lung function measurements

We performed spirometry according to ATS/ERS guidelines^17^^,^^18^ at ages 8, 11, 16 and 20 years in MAAS; 8, 15 and 24 years in ALSPAC; and 10, 18 and 26 years in IOW. Pre-bronchodilator FEV_1_ and FVC were recorded. To align data from the cohorts, we identified three epochs based on the availability of lung function: mid-school (8–10 years), adolescence (15–18 years) and early adulthood (20–26 years). We considered two transitions: from 8–10 to 15–18 (first) and from 15–18 to 20–26 years (second transition).

### Definition and derivation of spirometry phenotypes

Using pooled data, we performed regression analysis and estimated the regression residuals for FVC and FEV_1_/FVC adjusted for age, height, and race/ethnicity after stratification by sex. To make the cohorts more comparable, we used regression residuals instead of the GLI equation, as a recent study using data from 14 cohorts, including our cohorts, reported a high heterogeneity in GLI fit between age groups and cohorts.^19^ A more detailed rationale for using regression residuals is provided in Supplementary Appendix. The residual for each participant is the difference between his/her actual spirometry and expected spirometry. At each time-window (8–10, 15–18, 20–26 years), participants were assigned to one of three mutually exclusive spirometry phenotypes^8^:

(1) Normal: both FEV_1_/FVC and FVC residuals ≥10th percentile).
(2) Restrictive: FEV_1_/FVC residuals ≥10th percentile and FVC residuals <10th percentile; and
(3) Obstructive: FEV_1_/FVC residuals <10th percentile, independent of FVC residuals values.

The assignments were tested in three sensitivity analyses (Supplement).

### Statistical analysis

**Fig. 1:**
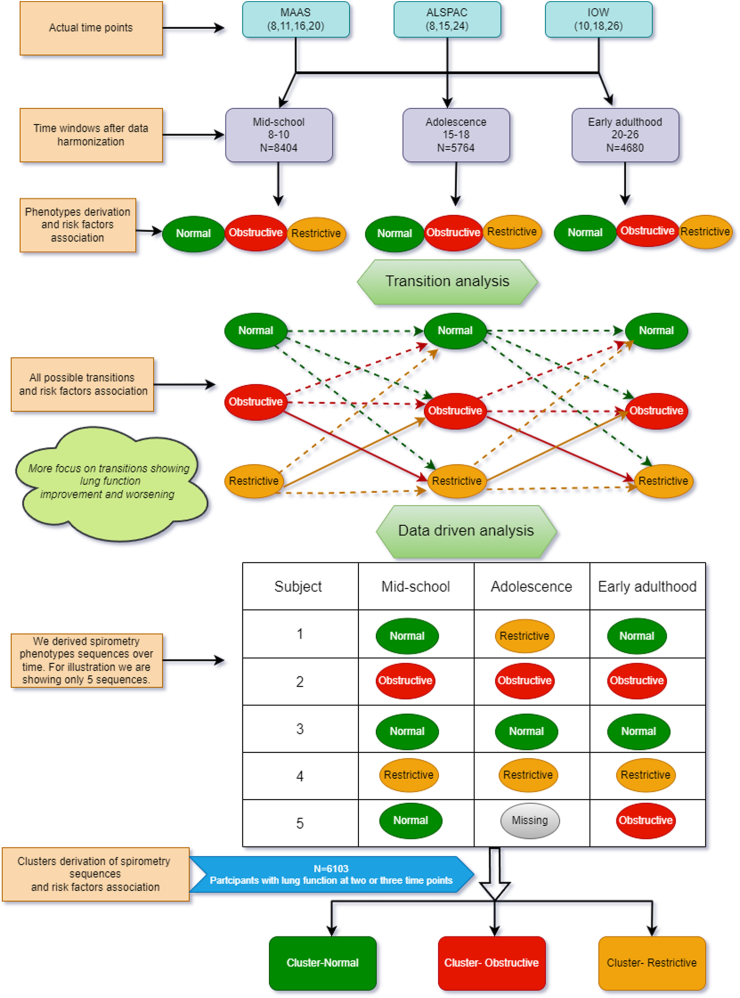
Flow chart of the different steps of data harmonization and statistical analysis.

Steps of statistical analysis are shown in Fig. 1. Approach to missing data is described in the Supplementary Appendix, and the missing data patterns are shown in Supplementary Figure S1.

#### Descriptive analyses

Descriptive analyses were performed to study overall, and time-specific prevalence of spirometry phenotypes and their associated risk factors.

#### Changes in spirometric obstruction and restriction over time

We considered two transitions: 8–10 to 15–18 years (first) and 15–18 to 20–26 years (second). To explore the changes in spirometry phenotypes over time, we created mutually exclusive patterns for obstructive and restrictive spirometry, with specific transitions defined as:

I) *Normal:* normal at time *t* and *t* + 1.
II) *For Obstructive phenotype:*
  1. Worsening: normal at time *t*, obstructive at *t* + 1.
  2. Improvement: obstructive at time *t*, normal at *t* + 1.
  3. Persistent obstruction: obstructive at time *t* and at *t* + 1.
III) *For Restrictive phenotype:*
  1. Worsening: normal at time *t*, restrictive at *t* + 1.
  2. Improvement: restrictive at time *t*, normal at *t* + 1.
  3. Persistent restriction: restrictive at time *t* and at *t* + 1.

#### Data driven analysis: patterns of change in spirometry phenotypes over time

We first derived sequences of spirometry phenotypes over time (Fig. 1). We then used Hierarchical Clustering coupled with optimal matching^20^ to derive clusters of the sequences. We used the Elbow and silhouette method to select optimal number of clusters.^20^

*Factors associated with spirometry clusters*: We used chi-square or Fisher exact tests for categorical variables, and analysis of variance (ANOVA) or Kruskal–Wallis's test for continuous variables. We used mixed-effect multinomial logistic regression models to study the association between spirometry clusters, outcomes, and risk factors; the results are reported as odds ratios (OR) with 95% confidence intervals (CI). To account for the between-study heterogeneity, we have added cohort as a random effect in all our regression models where appropriate. Data for selected risk factors were imputed using multiple imputations (Supplementary Appendix).^21^ We calculated variance inflation factors (VIF) for multivariate associations to examine multicollinearity; a VIF ≥5 was considered a cutoff point. All univariate and multivariate association analyses were performed in SAS 9.4. For cluster analysis, we used the R TraMineR package.^22^

The list of variables included in the multivariable analyses is shown in the Supplementary Appendix. Gestational age-adjusted birth weight (z-scores and centiles) was calculated using the tools developed by the International Fetal and Newborn Growth Consortium for the 21st Century.^23^ BMI for age z-scores and BMI for age categories (underweight: z-score <−1; normal: z-score ≥−1 and ≤1; overweight: z-score >1 and ≤2, obese: z-score >2) at the time of first lung function assessment were calculated based on the British 1990 Growth Reference.^24^

### Role of the funding source

The study’s funders had no role in the study design, data collection, analysis, interpretation, or report writing. The corresponding author had full access to all the data in the study and was ultimately responsible for deciding to submit it for publication.

## Results

We included 8404 participants at age 8–10, 5764 at age 15–18 and 4680 at age 20–26 years; the pooled and cohort-specific sample sizes are presented in Supplementary Table S1. Characteristics of study populations are shown in Supplementary Table S2.

### Spirometry phenotypes and their associated factors

Supplementary Figure S2 shows the observed proportions of spirometry phenotypes at each age in the overall study population and in each cohort. To validate our definitions, we calculated the means and standard deviations of FVC and FEV_1_/FVC Global Lung Function Initiative percent predicted values for each phenotype at each time (Supplementary Table S3). The mean value for FVC for restrictive phenotype ranged from 66.3% predicted to 78.7% predicted, and the mean FEV_1_/FVC for obstructive phenotype from 81.7% predicted to 85.7% predicted.

Supplementary Table S4 shows factors associated with spirometry phenotypes at all 3 time-points. Maternal smoking in pregnancy was significantly higher among participants with obstructive than those with normal phenotype at all times. The prevalence of pre-school wheezing, current wheezing, and current asthma diagnosis was significantly higher in obstructive phenotype. Subjects with restrictive phenotype were significantly more likely to be underweight at all time-points, and those with obstructive phenotype were more likely to be overweight or obese.

### Change in phenotypes over time and factors associated with change

**Fig. 2:**
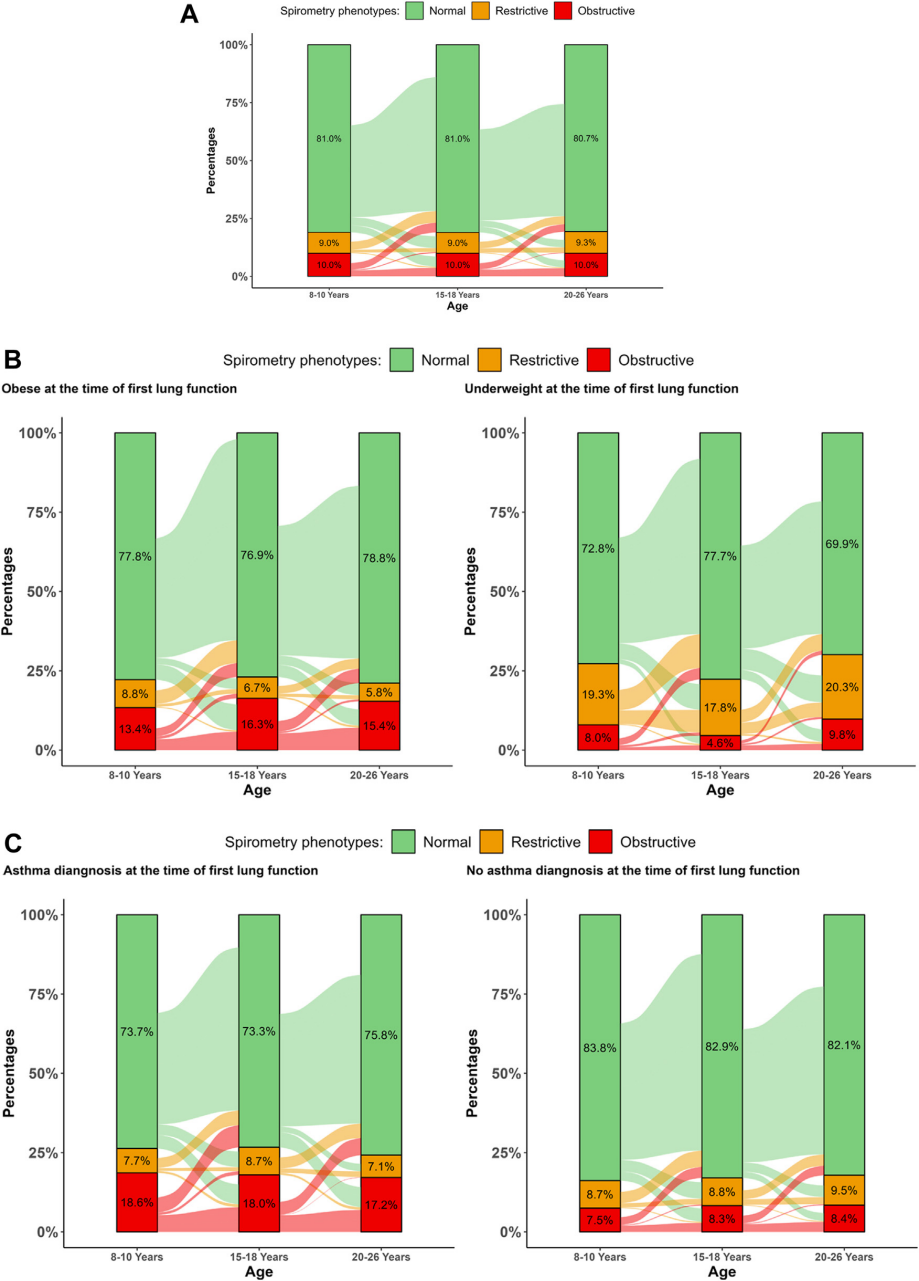
Alluvial plot shows the transitions/stability of restrictive and obstructive lung function over time for: A) Pooled data; B) Underweight and obese participants at the first lung function; and C) for participants with and without asthma diagnosis at the first lung function measurement. The percentages on bars show the prevalence of spirometry phenotypes at each time point. These graphs include all participants having lung function at any age.

Fig. 2A show the transitions between spirometry phenotypes across the time points. Normal phenotype was the most stable, with ∼87% of participants with normal spirometry maintaining normal lung function. The transition rates appeared higher at younger age. The most fluctuation was observed amongst those with restrictive phenotype, of whom 64.9% of (69.7% at first and 57.7% at second transition) transitioned to normal and 6.4% (7% at first and 5.3% at second transition) transitioned to the obstructive phenotype. Approximately 49% of those with obstructive phenotype moved to normal, and ∼5% moved to restrictive (5.9% at first and 3.7% at second transition), with ∼46% remaining obstructive. Similar patterns were observed for cohort-specific transitions (Supplementary Figure S3).

Fig. 2B and C shows the overall transitions stratified by BMI and asthma diagnosis. Underweight restrictive participants were less likely to transit to normal compared to obese (Overall transitions: 55.8% vs 74.1%); in contrast, obese obstructive participants were less likely to transit to normal compared to underweight (Overall transitions: 37.2% vs 50.0%). Higher transitions from normal to restrictive phenotype were observed in underweight participants (Overall transitions: normal to restrictive: 13.6%, normal to obstructive: 5.2%), but higher transitions from normal to obstructive phenotype were observed in obese participants (Overall transitions: normal to restrictive: 5.1%, normal to obstructive: 9.8%). Participants with asthma diagnosis were more likely to remain in the obstructive phenotype, and to transition from normal to obstructive (Overall transitions: 10.6% vs 5.2%) compared to those without asthma diagnosis.

### Improvement and worsening in spirometry obstructive and restrictive phenotypes

#### Obstructive spirometry

Supplementary Table S5 shows the prevalence of improvement, worsening, and persistent obstruction (∼6% population frequency for each phenotype at both transitions). The univariate factors associated with the change in phenotypes at two transitions are shown in Supplementary Table S6. Maternal smoking in pregnancy was significantly higher among those with worsening and persistent obstruction to adolescence (normal: 16.3%; worsening: 25.7%, improvers: 15.8%, persistent obstruction: 21.4%; p < 0.001). Early childhood wheeze, current wheeze, current asthma diagnosis and current allergic sensitization were significantly more prevalent among those with persistent obstruction and improvement at first transition. Obesity at the time of first lung function was less common among those with lung function improvement at first transition.

Supplementary Figure S4 shows the 10 most important factors of the change between obstructive phenotypes from multinomial logistic regression model. Maternal smoking during pregnancy and current asthma diagnosis increased the risk of worsening and had the most negative impact on improvement.

**Fig. 3:**
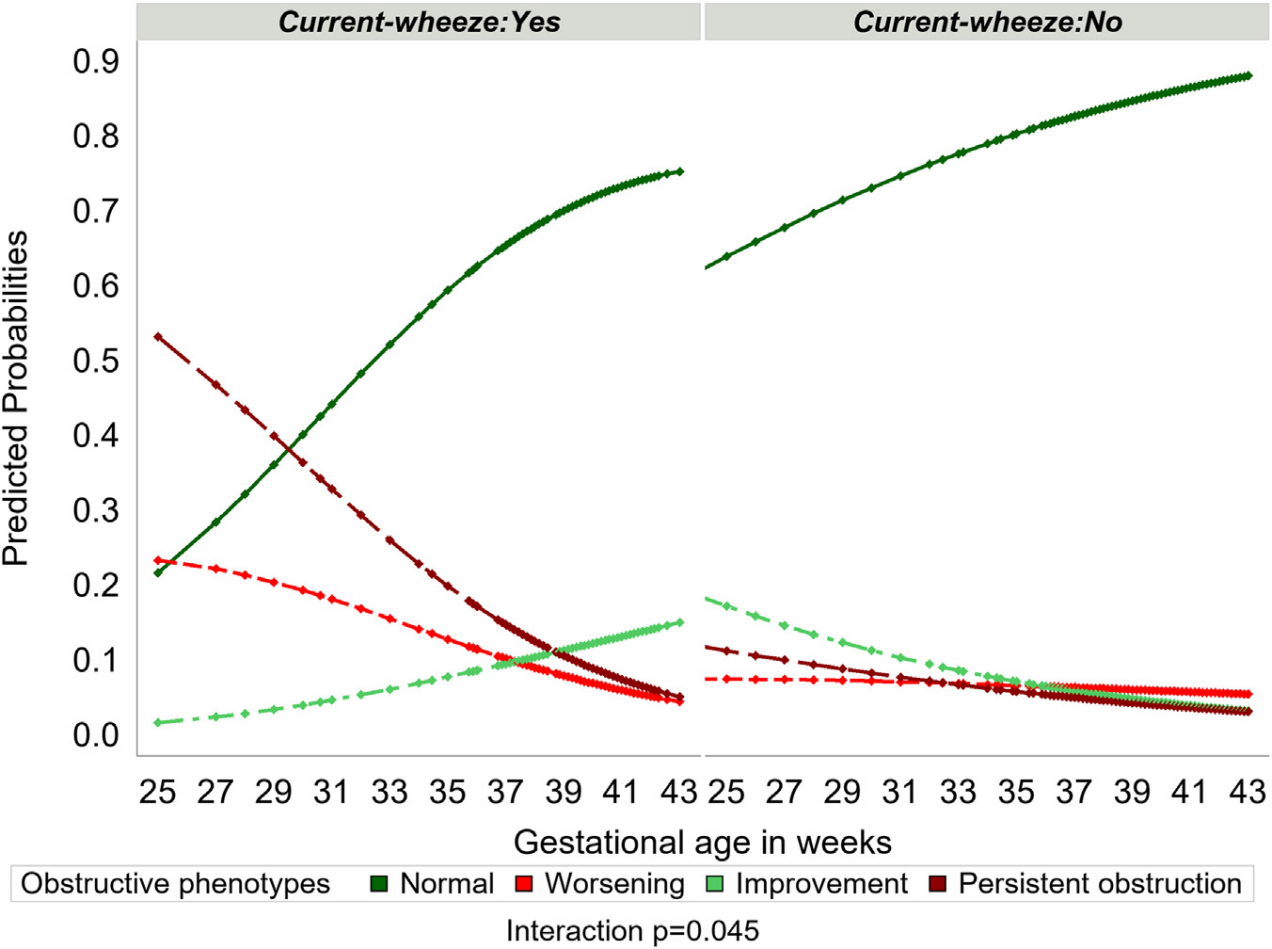
Predicted probabilities of obstructive phenotypes by gestational age and current wheeze. Points represent the predicted probability of each phenotype for each participant based on gestational age and current wheeze at the time of first lung function. The probabilities are estimated from a mixed-effect multinomial logistic regression model with gestational age, current wheeze, and its interaction as fixed effect covariates and cohort as a random effect. The outcome variable here is obstructive phenotype with four outcome categories, i.e., Normal, worsening, Improvement, and persistent obstruction.

To quantify the effect of gestational age and current wheeze on the probability of changes in obstructive spirometry, we fitted multinomial logistic regression with gestational age, current wheeze, and their interaction as covariates. A significant interaction was observed between gestational age and current wheeze (p = 0.045). The estimated probabilities are shown in Fig. 3. The probability of persistent obstruction was noticeably higher among participants with both lower gestational age and current wheeze, and decreased with increasing gestational age. Among current wheezers, the likelihood of improvement was higher among those with higher gestational age; conversely, among non-wheezers, the likelihood of improvement was higher among those with lower gestational age.

#### Restrictive spirometry

The population prevalence of worsening and improvement was ∼7% at the first transition and ∼6% at the second transition; the prevalence of persistent restriction was 2.4% at the first and 3.9% at the second transition (Supplementary Table S7).

The factors associated with the change in restrictive phenotypes at two transitions are shown in Supplementary Table S8. Birth weight and BMI were the only factors strongly associated with the change at the first transition, with mean birth weight centiles being the lowest among those with persistent restriction (p = 0.03; e.g., 14.9% of those with persistent restriction were underweight compared to 3.4% among normal). At the second transition, the only significant associate of change was BMI at the time of first lung function.

Importantly, for both phenotypes, the majority of those with improvement at the first transition remained having normal phenotype after second transition for participants with at least two lung function assessment, and participants with complete data (Table 1 and Supplementary Table S9).

**Table 1:** Frequencies and percentages of Lung function phenotypes sequences over time for participants with at least two lung function assessments (N = 6103).




Cluster indicator is also presented (please see the section on data-driven analyses).

### Data-driven analyses: clusters of spirometry phenotypes over time

Participants with at least two spirometry assessments were included in the cluster analysis (n = 6103). Comparisons between subjects included and excluded from the analyses are shown in Supplementary Table S10; frequency of maternal smoking during pregnancy was lower and of breastfeeding higher amongst included participants.

**Fig. 4:**
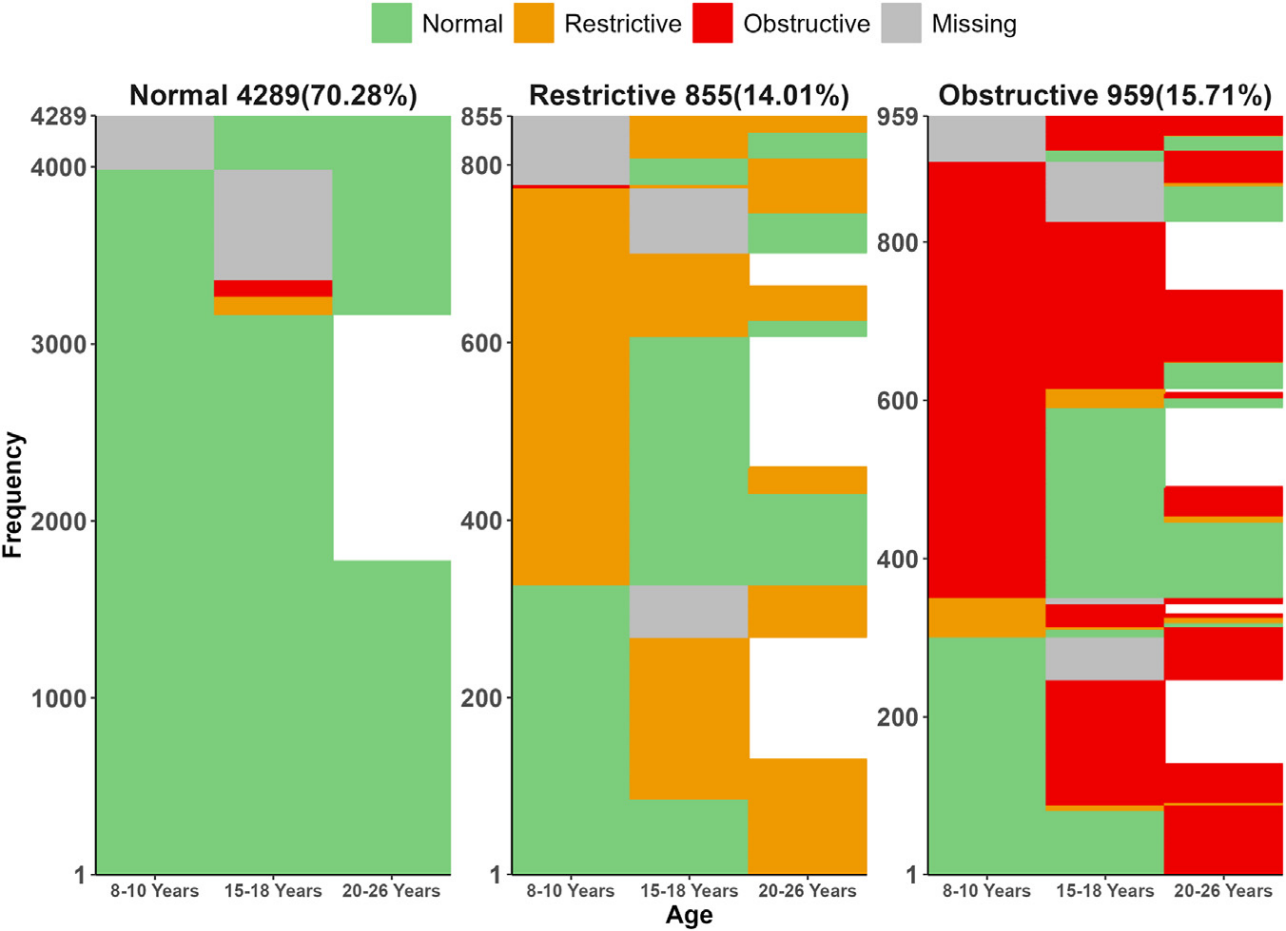
Clusters of the spirometry sequences over time (pooled data). After classifying each participant as normal, restrictive and obstructive at each time point, we clustered the individual participant's spirometry sequences. The grey colour represents missing values for the first or intermediate timepoints, whereas the white spaces represent loss to follow up. The normal cluster comprises those with Normal spirometry at all three time-points. In contrast, the Restrictive cluster comprises participants transitioning between normal and restrictive spirometry. The Obstructive cluster comprises participants mainly transitioning between normal and obstructive spirometry.

A three−cluster solution was selected as the optimal based on statistical fit (Supplementary Figure S5) and clinical interpretation. Clusters are depicted in Fig. 4 and were labelled as: (1) Normal: 4289 (70.27%); (2) Restrictive: 855 (14.01%); (3) Obstructive: 959 (15.72%). Clusters derived for participants with spirometry on all 3 time-points (n = 2739) were similar (Supplementary Figure S6). Furthermore, a similar three-cluster solution was evident when we clustered each cohort independently (Supplementary Figure S7). Interestingly, the cluster sizes for each cohort were consistent with the prevalence of the spirometry phenotypes, with MAAS having smaller restrictive cluster.

#### Factors associated with spirometry clusters

Multiple factors were associated with the obstructive cluster in the univariate analysis (Supplementary Table S11). Increase in gestational age [OR (95% CI): 0.94 (0.91–0.98); p = 0.003] and birth weight centiles [0.96 (0.94–0.99); p = 0.009] significantly decreases the odds of obstructive cluster membership. Maternal smoking during pregnancy, early childhood wheeze, BMI, current wheeze, current asthma diagnosis, current sensitization, and current parental smoking were all positively associated with the obstructive cluster membership.

BMI for age z-score [0.76 (0.70–0.83); p < 0.001] was negatively associated with restrictive cluster. Male sex [1.08 (1.01–1.16); p = 0.036], pet ownership during first year of life [1.11 (1.02–1.21); p = 0.015], and early childhood wheeze [1.11 (1.03–1.20); p < 0.001] were all positively associated with restrictive cluster.

Table 2 shows results from the mixed effect multiple multinomial logistic regression models (using both non-imputed and imputed data). The factors associated with obstructive and restrictive clusters in the univariate analyses were also associated with respective clusters in the multivariate analysis. Variance inflation factor (VIF) for both models was <5, and is shown for each associate in Supplementary Table S12.

**Table 2:** Adjusted odds ratios with 95% CI for spirometry clusters (Cluster-normal as a reference).

Risk factors	Cluster	Actual data Adjusted OR (95% CI)	p-value	Imputed data Adjusted OR (95% CI)	p-value
Male	Obstructive	0.90 (0.70, 1.15)	0.404	0.90 (0.77, 1.05)	0.176
	Restrictive	1.13 (0.87, 1.45)	0.354	1.21 (1.04, 1.41)	**0.013**
Maternal age	Obstructive	1.01 (0.98, 1.04)	0.265	1.01 (0.99, 1.02)	0.519
	Restrictive	1.01 (0.98, 1.04)	0.468	1.00 (0.98, 1.01)	0.741
Gestational age	Obstructive	0.96 (0.89, 1.03)	0.265	0.95 (0.91, 0.99)	**0.012**
	Restrictive	0.96 (0.88, 1.03)	0.267	0.98 (0.94, 1.02)	0.371
Birth weight centiles	Obstructive	0.97 (0.93, 1.01)	0.198	0.97 (0.95, 0.99)	**0.032**
	Restrictive	1.00 (0.99, 1.00)	0.997	0.99 (0.97, 1.020)	0.849
Parental asthma	Obstructive	1.17 (0.87, 1.55)	0.287	1.11 (0.93, 1.33)	0.248
	Restrictive	1.15 (0.84, 1.56)	0.390	0.87 (0.71, 1.06)	0.160
Maternal smoking during pregnancy	Obstructive	1.51 (1.11, 2.06)	**0.009**	1.37 (1.14, 1.65)	**<0.001**
	Restrictive	0.91 (0.62, 1.32)	0.604	0.89 (0.72, 1.11)	0.314
Breast feeding first six months	Obstructive	0.98 (0.71, 1.34)	0.877	1.18 (0.96, 1.44)	0.101
	Restrictive	0.91 (0.64, 1.27)	0.572	1.01 (0.82, 1.24)	0.913
Pet ownership first year of life^a^	Obstructive	1.11 (0.86, 1.41)	0.418	1.02 (0.87, 1.19)	0.778
	Restrictive	1.35 (1.03, 1.75)	**0.026**	1.29 (1.09, 1.51)	**0.002**
Early childhood wheeze^b^	Obstructive	1.79 (1.39, 2.29)	**<0.001**	1.86 (1.60, 2.17)	**<0.001**
	Restrictive	1.32 (1.01, 1.71)	**0.038**	1.31 (1.12, 1.54)	**<0.001**
BMI z-score at first LF^c^	Obstructive	0.92 (0.74, 1.14)	0.461	1.00 (0.87, 1.14)	0.955
	Restrictive	0.83 (0.66, 1.03)	0.098	0.68 (0.59, 0.78)	**<0.001**
Current wheeze at first LF	Obstructive	1.88 (1.23, 2.87)	**0.003**	1.34 (1.05, 1.71)	**0.016**
	Restrictive	1.10 (0.64, 1.90)	0.719	1.06 (0.79, 1.41)	0.697
Current asthma diagnosis at first LF	Obstructive	1.07 (0.70, 1.63)	0.760	1.47 (1.15, 1.86)	**0.002**
	Restrictive	0.62 (0.36, 1.06)	0.078	0.83 (0.62, 1.11)	0.204
Current sensitization at first LF	Obstructive	1.45 (1.08, 1.93)	**0.012**	1.25 (1.04, 1.51)	**0.017**
	Restrictive	1.10 (0.78, 1.53)	0.576	1.01 (0.82, 1.24)	0.906
Current Parental smoking at first LF	Obstructive	1.21 (0.93, 1.57)	0.146	1.16 (0.98, 1.36)	0.075
	Restrictive	0.92 (0.69, 1.23)	0.577	0.85 (0.71, 1.02)	0.081

LF: lung function.

Where adjustment is done for all the factors, i.e., all factors included in the model.

Variance inflation factor (VIF): VIF for both models were less than 5. VIF for each risk factors are given in Supplementary Table S12. p-values < 0.05 are shown in bold.

a Pet inside the house or contact with pets most of the time in first year of life.

b Current wheeze at age five or below years.

c Based on the British 1990 Growth Reference.

#### Probabilities of cluster membership stratified by selected risk factors

The probability of restrictive cluster membership decreased with increasing BMI (Supplementary Figure S8). After stratifying by asthma diagnosis, for both asthmatics and non-asthmatics, the probability of restrictive cluster membership decreases with increasing BMI. In contrast, the probability of obstructive cluster membership increased with increasing BMI. Of note, the effect of BMI on obstructive cluster membership was comparatively stronger in those with current asthma diagnosis than those without (Supplementary Figure S9).

Supplementary Figure S10 shows the predicted probabilities of cluster membership by BMI and wheeze phenotypes. The probability of normal cluster membership was lower for the underweight participants and those with persistent wheezing, while the probability of obstructive cluster was higher for those with higher BMI and persistent wheezing. The probability of restrictive cluster was only affected by BMI, where lower BMI resulted in a higher probability of restrictive cluster membership.

## Discussion

Using data from three population-based cohorts with repeated measures of lung function we estimated the prevalence of spirometric obstruction and restriction, and their transitions from school-age to early adulthood. Maternal smoking during pregnancy, early wheeze, higher BMI, current wheeze, current asthma diagnosis, current sensitization, and current parental smoking were associated with higher prevalence of obstructive phenotype at each age. Spirometric restriction was the most variable phenotype with the highest transition rate, and was markedly higher among underweight participants. The worsening and improvement in both phenotypes were observed at both transition points, with substantial proportion of those with obstruction and restriction moving to normal phenotype (up to 50%), and some of those with normal lung function (∼5%) developing impairment. Importantly, the improvement appeared stable; among restrictive participants, ∼48% transited to normal lung function at the first transition and continued normal on the next transition, compared to 13.4% who transitioned back to restrictive phenotype. Similarly, 33.5% of obstructive subjects transited to normal lung function and remained normal, compared to 12.7% who transitioned back to obstructive. Of note, transition rates were higher at young age, suggesting that any potential intervention should start early in childhood. In the analysis of change over time, maternal smoking during pregnancy markedly increased the odds of worsening in lung function and decreased the odds of improvement. Early wheeze and presence and persistence of asthma symptoms and asthma diagnosis was associated with persistent obstruction or becoming obstructive. Underweight restrictive and obese obstructive participants were less likely to transition to normal. Significant associate of worsening in restrictive phenotypes was lower BMI at the first lung function assessment. Among current wheezers, the highest probability of persistent obstruction was for those with low gestational age, whereas the highest improvement was observed among those with higher gestational age.

Several studies reported the prevalence of spirometry phenotypes in childhood and early adulthood using different phenotype definitions.^8^^,^^9^ We applied the definition derived by Voraphani et al.^8^ In our study, the mean predicted FVC in restrictive phenotypes ranged from 66.3% to 78.7%, consistently lower than 85.5% (the cut-off reported as the most favourable trade-off between sensitivity and specificity for diagnosing lung function restriction^25^). We observed the overall prevalence of obstructive phenotypes of ∼10%, and that of restrictive ∼9% at all ages, which is consistent with previous findings.^8^^,^^9^ In all studies, there was no change in point prevalence of spirometric obstruction and restriction with increasing age.^9^ However, our study offered an opportunity to investigate changes in spirometry impairments within individuals and demonstrated that a substantial proportion of participants with impairments transited to normal spirometry. We focussed on clinically relevant ventilatory defects (obstructive and restrictive phenotypes) to understand lung function catch-up and worsening. In our study, multiple factors were associated with changes in obstructive spirometry. A higher probability of persistent obstruction was observed among those with low gestational age and current wheeze, and maternal smoking during pregnancy was higher among participants with persistent obstruction and worsening from normal to obstructive spirometry. This is consistent with findings that the developing foetal lung is susceptible to the effects of *in-utero* tobacco smoke exposure, with altered lung function in early infancy which is long-lasting and affects subsequent lung function trajectory and lung health.^26^^,^^27^ In our study, early and current wheeze, current asthma diagnosis and allergic sensitization were higher among those with persistent obstruction. Improvement was also more likely in those with current wheeze and asthma diagnosis, which is important and indicates that patients with these conditions have a potential for improvement. The only two risk factors associated with a change in spirometric restriction were birth weight centiles and BMI at the time of first lung function. Low birth weight was associated with persistent restriction, which might be explained by restricted intrauterine growth influencing airway and parenchymal development, potentially causing dysanapsis (disproportionate scaling of airway dimensions to lung volume) and affecting subsequent lung function and health.^28^^,^^29^

In recent years, several studies described lifetime trajectories of FEV_1_ or FEV_1_/FVC and their predictors using data-driven methods.^30^, ^31^, ^32^, ^33^, ^34^, ^35^, ^36^, ^37^ Few studies applied modelling to childhood lung function, as repeated spirometry is relatively rarely available in this age group.^30^, ^31^, ^32^^,^^36^ Analyses identified between two^31^ and four^30^^,^^32^^,^^36^ trajectories from school-age into adolescence, depicted by parallel lines of apparently stable lung function, often interpreted as “tracking” through childhood.^36^^,^^38^ Unbiased analyses to date revealed no evidence of clusters of children with improving or declining lung function.^6^ However, clinical experience and inspection of within-individual trajectories suggests that lung function improves in some, and declines in others. One previous study, which modelled repeated measures of specific airway resistance rather than spirometry, reported that children with persistent wheeze, frequent exacerbations and early atopy are at risk of lung function decline between ages 3 and 11 years.^39^ Recently, Wang et al. reported the analysis using data-driven Markovian model which identified five latent states of lung function (based on FEV_1_ z-scores) at three cross-sectional points from childhood to early adulthood (very low, low, normal, high and very high). The authors defined “catch-up” and “growth failure” *a posteriori*, for subsequent hypothesis testing (those moving from the low/very low to normal/high/very high states were assigned as “catch-up” group, and those moving from normal/high/very high to the low/very low states to a “growth failure”). In our study, we reported the longitudinal transition pattern, i.e., improvement and worsening, using clinically relevant phenotypes of impaired lung function (obstructive and restrictive) based on measurement of both FVC and FEV_1_/FVC. Although these two analyses addressed different questions, used different measures of lung function, different definitions of changes in lung function over time, and different approaches to data analysis, the findings demonstrated that broadly similar early-life risk factors were associated with features which most clinicians would associate with improvement or worsening of lung function.

Previous analysis in one of our birth cohorts (ALSPAC) suggested that catch-up growth is possible around puberty and is associated with later onset and higher velocity of pubertal growth.^40^ Given the relationships between the early-onset of puberty with child’s and maternal obesity and gestational weight gain,^41^ it has been suggested that a combination of interventions to reduce childhood obesity and obesity in pregnancy may have substantial impact on life-long health.^42^

Our unbiased analysis identified three clusters of spirometry phenotypes over time (normal, restrictive, and obstructive), but similar to previous analyses of childhood lung function, no clusters characterised by improvement/decline were detected (reviewed in^6^). Results of our frequentist analyses, which confirmed that improvement and worsening in spirometry phenotypes does occur but is rare at the population level, suggest that studies with much larger sample sizes are required to identify such patterns in data-driven analyses.^6^^,^^11^ The restrictive cluster in our analysis included those transitioning between normal and restrictive, and obstructive cluster those who were transitioning between normal and obstructive. Maternal smoking during pregnancy, early wheeze, current wheeze, current BMI, current asthma diagnosis, current allergic sensitization and current parental smoking were all positively associated with the obstructive cluster. The effect of BMI on obstructive cluster membership was stronger in those with asthma diagnosis, emphasising the importance of reducing the weight among overweight/obese children with asthma. The unbiased analysis facilitated more precise investigation of factors associated with restrictive cluster, and identified male gender and pet ownership in infancy as associates with the restrictive cluster (in addition to the strong effect of BMI).

Several limitations of the current study should be noted. One of the common limitations of analysing data on lung function from multiple cohorts is that there may exists systematic bias in recording spirometry (including the use of different equipment and operators). We used internal reference, i.e., regression residuals, to provide an alternative approach and account for potential bias in GLI based scores), but the use of regression residuals-based phenotypes limited the generalizability of our findings. Since we used pooled individual-level data from three cohorts, there might exist between study heterogeneity. To address this, we used cohort as a random effect in all our regression models and residual derivation. Furthermore, we derived the cluster in each cohort independently and the resulting cohorts were consistent to that of pooled data. Another limitation of our study is that the population is not ethnically diverse (>95% of participants are of white European ancestry). Our results are therefore not transferable to other ethnic groups. Early life pulmonary/airway function tests were not performed, which limits the inference to the potential role of preschool lung function. Like most other studies investigating longitudinal lung function, we used baseline (i.e., pre-bronchodilator) spirometry. Postbronchodilator spirometry may be a more accurate predictor of subsequent respiratory diseases such as COPD,^43^ but longitudinal data on post-bronchodilator lung function during childhood is rarely collected in birth cohort studies. Collecting such information in ongoing or future studies could prove very valuable. Finally, our current study is exploratory in nature. Although multiple early-life risk factors, and those contemporaneous with the first assessment of lung function were considered, residual confounding, by e.g., diet or physical activity, socioeconomic and unexplored risk factors, may be important.

In conclusion, our results provide evidence that within-individual changes in lung function over time (including improvement and worsening) do occur during childhood, and that a substantial proportion of children with lung function impairment in early school age (between one third in obstructive phenotype and a half in restricted phenotype) improve and achieve normal and stable lung function to early adulthood. Conversely, <5% of those with normal lung function in early school age worsening to early adulthood. Analysis of the factors associated with improvement results suggest that efforts to reduce foetal growth restriction and premature birth, maintain optimal weight during childhood, and control maternal smoking during pregnancy may significantly reduce the prevalence of spirometry obstruction and restriction and declining lung function.

## Contributors

A.C., G.R., A.S., A.U., R.G., and J.W.H. conceived and planned the study and wrote the manuscript. A.C., H.A., C.S.M., S.T., J.W.T., A.S., and G.R. were responsible for the acquisition of the financial support for the project leading to this publication. A.U., R.G., S.H., and S.F. developed and applied statistical, mathematical, and computational techniques to analyze and synthesize data. A.U., R.G., S.H., and S.F. were responsible for the annotation and maintenance of the research data (including software code for interpreting the data). A.U., R.G., and S.F. were responsible for visualization/data presentation. A.C., H.A., C.S.M., S.T., J.W.T., L.L., A.S., and G.R. conducted investigation process, L.L. performed longitudinal lung function measurements in MAAS. All authors contributed to the interpretation of the results. All authors provided critical feedback and helped shape the research, analysis, and manuscript. A.U., R.G., S.H., and S.F. directly accessed and verified the underlying data reported in the manuscript.

## Data sharing statement

De-identified participant data and data dictionary are available to others; each cohort has its own policies and procedure to share the data and can be contacted for data request.

## Declaration of interests

All authors have completed the ICMJE uniform disclosure form. Dr Custovic reports personal fees outside the submitted work from Sanofi, Stallergenes Greer, AstraZeneca, GSK, and La Roche-Posay. Dr Custovic also reported grants or contracts from MRC, EPSRC, and Welcome Trust and declared a leadership role in the World Allergy Organization. Clare Murray reports personal fees from Sanofi and GSK. Graham Roberts declared a leadership role in the British Society for Allergy & Clinical Immunology (BSACI). Angela Simpson reported grants or contracts from NIHR. Other authors declare no conflict of interest.

## References

[1] Agusti A., Noell G., Brugada J., Faner R. (2017). Lung function in early adulthood and health in later life: a transgenerational cohort analysis. Lancet Respir Med.

[2] Lange P., Celli B., Agusti A. (2015). Lung-function trajectories leading to chronic obstructive pulmonary disease. N Engl J Med.

[3] Cuttica M.J., Colangelo L.A., Dransfield M.T. (2018). Lung function in young adults and risk of cardiovascular events over 29 years: the CARDIA study. J Am Heart Assoc.

[4] Cheng Y.J., Chen Z.G., Yao F.J., Liu L.J., Zhang M., Wu S.H. (2022). Airflow obstruction, impaired lung function and risk of sudden cardiac death: a prospective cohort study. Thorax.

[5] Guerra S., Sherrill D.L., Venker C., Ceccato C.M., Halonen M., Martinez F.D. (2010). Morbidity and mortality associated with the restrictive spirometric pattern: a longitudinal study. Thorax.

[6] Custovic A., Fontanella S. (2023). Evolution of lung function within individuals: clinical insights and data-driven methods. Am J Respir Crit Care Med.

[7] Mannino D.M., McBurnie M.A., Tan W. (2012). Restricted spirometry in the burden of lung disease study. Int J Tuberc Lung Dis.

[8] Voraphani N., Stern D.A., Zhai J. (2022). The role of growth and nutrition in the early origins of spirometric restriction in adult life: a longitudinal, multicohort, population-based study. Lancet Respir Med.

[9] Wang G, Hallberg J., Charalampopoulos D. (2021). Spirometric phenotypes from early childhood to young adulthood: a Chronic Airway Disease Early Stratification study. Erj Open Res.

[10] Huang L., Wang S.T., Kuo H.P. (2021). Effects of obesity on pulmonary function considering the transition from obstructive to restrictive pattern from childhood to young adulthood. Obes Rev.

[11] Wang G., Hallberg J., Faner R. (2023). Plasticity of individual lung function states from childhood to adulthood. Am J Respir Crit Care Med.

[12] Custovic A., Simpson B.M., Murray C.S., Lowe L., Woodcock A. (2002). The national asthma campaign Manchester asthma and allergy study. Pediatr Allergy Immunol.

[13] Golding J., Pembrey M., Jones R. (2001). ALSPAC-the avon longitudinal study of parents and children. I. Study methodology. Paediatr Perinat Epidemiol.

[14] Kurukulaaratchy R.J., Fenn M., Twiselton R., Matthews S., Arshad S.H. (2002). The prevalence of asthma and wheezing illnesses amongst 10-year-old schoolchildren. Respir Med.

[15] Custovic A., Ainsworth J., Arshad H. (2015). The study team for early life asthma research (STELAR) consortium 'Asthma e-lab': team science bringing data, methods and investigators together. Thorax.

[16] Haider S., Granell R., Curtin J. (2022). Modeling wheezing spells identifies phenotypes with different outcomes and genetic associates. Am J Respir Crit Care Med.

[17] Miller M.R., Hankinson J., Brusasco V. (2005). Standardisation of spirometry. Eur Respir J.

[18] Beydon N., Davis S.D., Lombardi E. (2007). An official American Thoracic Society/European Respiratory Society statement: pulmonary function testing in preschool children. Am J Respir Crit Care Med.

[19] Wang G., Hallberg J., Charalampopoulos D. (2021). Spirometric phenotypes from early childhood to young adulthood: a chronic airway disease early stratification study. ERJ Open Res.

[20] Kaufman L., Rousseeuw P.J., ProQuest (2005).

[21] Yuan Y. (2011). Multiple imputation using SAS software. J Stat Softw.

[22] Gabadinho A., Ritschard G., Muller N.S., Studer M. (2011). Analyzing and visualizing state sequences in R with TraMineR. J Stat Softw.

[23] Villar J., Ismail L.C., Victora C.G. (2014). International standards for newborn weight, length, and head circumference by gestational age and sex: the Newborn Cross-Sectional Study of the INTERGROWTH-21st Project. Lancet.

[24] Vidmar S.I., Cole T.J., Pan H.Q. (2013). Standardizing anthropometric measures in children and adolescents with functions for egen: update. Stata J.

[25] Myrberg T., Lindberg A., Eriksson B. (2022). Restrictive spirometry versus restrictive lung function using the GLI reference values. Clin Physiol Funct Imaging.

[26] Hayatbakhsh M.R., Sadasivam S., Mamun A.A., Najman J.M., Williams G.M., O'Callaghan M.J. (2009). Maternal smoking during and after pregnancy and lung function in early adulthood: a prospective study. Thorax.

[27] McEvoy C.T., Spindel E.R. (2017). Pulmonary effects of maternal smoking on the fetus and child: effects on lung development, respiratory morbidities, and life long lung health. Paediatr Respir Rev.

[28] den Dekker H.T., Jaddoe V.W.V., Reiss I.K., de Jongste J.C., Duijts L. (2018). Fetal and infant growth patterns and risk of lower lung function and asthma the generation R study. Am J Resp Crit Care.

[29] Vameghestahbanati M., Sack C., Wysoczanski A. (2023). Association of dysanapsis with mortality among older adults. Eur Respir J.

[30] Belgrave D.C.M., Granell R., Turner S.W. (2018). Lung function trajectories from pre-school age to adulthood and their associations with early life factors: a retrospective analysis of three population-based birth cohort studies. Lancet Respir Med.

[31] Berry C.E., Billheimer D., Jenkins I.C. (2016). A distinct low lung function trajectory from childhood to the fourth decade of life. Am J Resp Crit Care.

[32] Karmaus W., Mukherjee N., Janjanam V.D. (2019). Distinctive lung function trajectories from age 10 to 26 years in men and women and associated early life risk factors - a birth cohort study. Respir Res.

[33] Bui D.S., Lodge C.J., Burgess J.A. (2018). Childhood predictors of lung function trajectories and future COPD risk: a prospective cohort study from the first to the sixth decade of life. Lancet Respir Med.

[34] McGeachie M.J., Yates K.P., Zhou X. (2016). Patterns of growth and decline in lung function in persistent childhood asthma. N Engl J Med.

[35] Weber P., Menezes A.M.B., Goncalves H. (2020). Characterisation of pulmonary function trajectories: results from a Brazilian cohort. ERJ Open Res.

[36] Sanna F., Locatelli F., Sly P.D. (2022). Characterisation of lung function trajectories and associated early-life predictors in an Australian birth cohort study. ERJ Open Res.

[37] Dharmage S.C., Bui D.S., Walters E.H. (2023). Lifetime spirometry patterns of obstruction and restriction, and their risk factors and outcomes: a prospective cohort study. Lancet Respir Med.

[38] Bardsen T., Roksund O.D., Benestad M.R. (2022). Tracking of lung function from 10 to 35 years after being born extremely preterm or with extremely low birth weight. Thorax.

[39] Belgrave D.C., Buchan I., Bishop C., Lowe L., Simpson A., Custovic A. (2014). Trajectories of lung function during childhood. Am J Respir Crit Care Med.

[40] Mahmoud O., Granell R., Tilling K. (2018). Association of height growth in puberty with lung function. a longitudinal study. Am J Respir Crit Care Med.

[41] Zhou J., Zhang F., Zhang S. (2022). Maternal pre-pregnancy body mass index, gestational weight gain, and pubertal timing in daughters: a systematic review and meta-analysis of cohort studies. Obes Rev.

[42] Bush A. (2021). Growing, growing, gone: the double whammy of early deprivation and impaired evolution of lung function. Am J Respir Crit Care Med.

[43] Fortis S., Eberlein M., Georgopoulos D., Comellas A.P. (2017). Predictive value of prebronchodilator and postbronchodilator spirometry for COPD features and outcomes. BMJ Open Respir Res.

