# Supplementary material for: Obstructive and restrictive spirometry from school age to adulthood: three birth cohort studies
Source: eClinicalMedicine. 2023 Dec 12;67:102355. doi: 10.1016/j.eclinm.2023.102355 (PMC10758747; doi:10.1016/j.eclinm.2023.102355)
Supplement: Supplementary Appendix [file mmc1.docx]

**Obstructive and restrictive spirometry from school age to adulthood: Three birth cohort studies**

**Supplementary Appendix**

Anhar Ullah^1,2§^, Raquel Granell^3§^, Sadia Haider^1,2^, Lesley Lowe^4^, Sara Fontanella^1,2^, Hasan Arshad^5,6,7^, Clare S Murray^4^, Steve Turner^8,9^, John W Holloway^5,6^, Angela Simpson^4*^, Graham Roberts^5,6,7*^, Adnan Custovic^1,2*^

on behalf of STELAR/UNICORN^10^ investigators

^§^Equal contribution

*Joint senior authors

^1^National Heart and Lung Institute, Imperial College London, UK

^2^NIHR Imperial Biomedical Research Centre, London, UK

^3^Department of Population Health Sciences, Bristol Medical School, University of Bristol, UK

^4^Division of Infection, Immunity and Respiratory Medicine, School of Biological Sciences, Faculty of Biology, Medicine and Health, University of Manchester, Manchester Academic Health Science Centre, U.K.

^5^Human Development and Health, Faculty of Medicine, University of Southampton, Southampton, U.K.

^6^NIHR Southampton Biomedical Research Centre, University Hospitals Southampton NHS Foundation Trust, Southampton, U.K.

^7^David Hide Asthma and Allergy Research Centre, Isle of Wight, UK

^8^Royal Aberdeen Children's Hospital NHS Grampian Aberdeen, AB25 2ZG, UK

^9^Child Health, University of Aberdeen, Aberdeen, UK

# ^10^STELAR/UNICORN investigators:

Professor John Ainsworth, School of Health Sciences, The University of Manchester

Dr Philip Couch, School of Health Sciences, The University of Manchester

Professor Paul Cullinan, Imperial College London

Professor Graham Devereux, Clinical Sciences, Liverpool School of Tropical Medicine

Professor Ashley Woodcock, The University of Manchester

Contents

[SUPPLEMENTARY METHODS 2](#_Toc143699781)

[The Manchester Asthma and Allergy Study (MAAS) 2](#_Toc143699782)

[The Avon Longitudinal Study of Parents and Children (ALSPAC) 3](#_Toc143699783)

[The Isle of Wight (IOW) cohort 3](#_Toc143699784)

[Spirometry 4](#_Toc143699785)

[MAAS 4](#_Toc143699786)

[ALSPAC 4](#_Toc143699787)

[IOW 4](#_Toc143699788)

[Definitions of variables (demographic, exposures and outcomes) 4](#_Toc143699789)

[Data-driven phenotype allocation 4](#_Toc143699790)

[STATISTICAL ANALYSIS 5](#_Toc143699791)

[Definition and derivation of spirometry phenotypes: Sensitivity analyses 5](#_Toc143699792)

[Descriptive analyses 5](#_Toc143699793)

[Data driven analysis: Patterns of change in spirometry phenotypes over time 5](#_Toc143699794)

[Missing data and imputation 5](#_Toc143699795)

[The list of variables included in the analyses of the associates of spirometry clusters 7](#_Toc143699796)

[SUPPLEMENTARY RESULTS 8](#_Toc143699797)

[DEMOGRAPHICS, PREVALENCE OF SPIROMETRY PHENOTYPES, AND THEIR ASSOCIATES 8](#_Toc143699798)

[CHANGE IN SPIROMETRY PHENOTYPES OVER TIME AND THE ASSOCIATES OF CHANGE 16](#_Toc143699799)

[IMPROVEMENT AND DECLINE IN SPIROMETRY PHENOTYPES OVER TIME 19](#_Toc143699800)

[DATA-DRIVEN ANALYSES TO IDENTIFY CLUSTERS OF THE SPIROMETRY SEQUENCES OVER TIME 27](#_Toc143699801)

# SUPPLEMENTARY METHODS

## The Manchester Asthma and Allergy Study (MAAS)

MAAS is an unselected birth cohort study established in 1995 in Manchester, UK^1^. It consists of a mixed urban-rural population within 50 square miles of South Manchester and Cheshire, located within the maternity catchment area of Wythenshawe and Stepping Hill Hospitals. All pregnant women were screened for eligibility at antenatal visits (8-10^th^ week of pregnancy). Of the 1499 couples who met the inclusion criteria (≤10 weeks of pregnancy, maternal age ≥18 years, and questionnaire and skin prick data test available for both parents), 288 declined to take part in the study and 27 were lost to follow-up between recruitment and the birth of a child. A total of 1184 children were born into the study between February 1996 and April 1998. They were followed prospectively for 20 years and attended follow-up clinics for assessments, which included lung function measurements, skin prick testing, biological samples (serum, plasma and urine), and questionnaire data collection. The study was approved by the North-West – Greater Manchester East Research Ethics Committee.

We capitalized on a unique feature of the health care system in the UK in that, General practitioners (GPs) maintain primary care records of all health care encounters of their patients, including hospital admission, and outpatient appointments. A trained paediatrician extracted and transcribed data from GP-held medical records including AD diagnosis and prescriptions for topical treatments. Timing, type of visit, symptoms, indication and prescriptions for each encounter were noted. A total of 987 participants provided informed consent for medical data collection. We reviewed 925 study participants due to GPs' lack of response for data collection or participants moving away. Nine of these were partially accessed due to missing paper or electronic records and were excluded.

Data on lower respiratory tract infections (LRTI), hospital admissions, bronchiolitis, and RSV-positive bronchiolitis were extracted from electronic and paper-based primary care medical records, including emergency department admissions, and hospital admissions. Age in days at the time of each event was documented. This data was available from birth to age 8-10 years.

## The Avon Longitudinal Study of Parents and Children (ALSPAC)

ALSPAC is a birth cohort study established in 1991 in Avon, UK^2-4^. Pregnant women with expected dates of delivery 1^st^ April 1991 to 31^st^ December 1992 were invited to take part in the study. The initial number of pregnancies enrolled is 14,541. Of these initial pregnancies, there was a total of 14,676 foetuses, resulting in 14,062 live births and 13,988 children who were alive at 1 year of age.

When the oldest children were approximately 7 years of age, an attempt was made to bolster the study with eligible cases who had failed to join originally. As a result, when considering variables collected from the age of seven onwards (and potentially abstracted from obstetric notes) there are data available for more than the 14,541 pregnancies mentioned above. The number of new pregnancies not in the initial sample (known as Phase I enrolment) that are currently represented on the built files and reflecting enrolment status at the age of 24 is 913 (456, 262 and 195 recruited during Phases II, III and IV respectively), resulting in an additional 913 children being enrolled. The phases of enrolment are described in more detail in the cohort profile paper and its update. The total sample size for analyses using any data collected after the age of seven is therefore 15,454 pregnancies, resulting in 15,589 foetuses. Of these 14,901 were alive at 1 year of age.

Study data were collected and managed using REDCap (Research Electronic Data Capture) electronic data capture tools^5^ hosted at University of Bristol. Ethical approval for the study was obtained from the ALSPAC Ethics and Law Committee and the Local Research Ethics Committees. Informed consent for the use of data collected via questionnaires and clinics was obtained from participants following the recommendations of the ALSPAC Ethics and Law Committee at the time. The study website contains details of available data through a fully searchable data dictionary and variable search tool: <http://www.bristol.ac.uk/alspac/researchers/our-data/>

We are extremely grateful to all the families who took part in this study, the midwives for their help in recruiting them, and the whole ALSPAC team, which includes interviewers, computer and laboratory technicians, clerical workers, research scientists, volunteers, managers, receptionists and nurses.

## The Isle of Wight (IOW) cohort

IOW is an unselected birth cohort study established in 1989 on the Isle of Wight, UK^6-8^. After the exclusion of adoptions, perinatal deaths, and refusal for follow-up, written informed consent was obtained from parents to enrol 1,456 newborns (of 1536 born between 1^st^ January 1989 and 28^th^ February 1990). Follow-up-up assessments were conducted to 26 years of age to prospectively study the development of asthma and allergic diseases. At each follow-up, validated questionnaires were completed by the parents. Ethics approvals were obtained from the Isle of Wight Local Research Ethics Committee (now named the National Research Ethics Service, NRES Committee South Central – Southampton B) at recruitment and for the subsequent follow-ups.

The IOW research team are grateful to all the participants and their families for their support over the years and also to the many fellow researchers who have contributed to the cohort's follow up.

## Spirometry

### MAAS

Spirometry was performed at ages 8, 11, 16 and 20 years according to American Thoracic Society/European Respiratory Society guidelines^9,10^ using a Lilly pneumotachograph system with animated incentive software (Jaeger, Germany). For home visits, we used a flow turbine spirometer (Micro Medical, UK). Subjects were asked to inhale to total lung capacity (TLC), then instructed to perform a forced expiration, through a mouthpiece, to residual volume (RV). The test was repeated at intervals of 30 seconds until 3 technically acceptable traces were obtained. Forced expiratory volume in one second (FEV_1_) and Forced vital capacity (FVC) were recorded and the data expressed as FEV_1_ % predicted and FEV_1_/FVC ratio. Short-acting β2-agonists were withheld for at least four, and long-acting for at least 24 hours prior to testing. Participants were symptom-free at the time of assessment.

### ALSPAC

Spirometry tests were conducted at 8^1/2^, 15 and 24 years according to American Thoracic Society/European Respiratory Society guidelines^9,10^ using a Vitalograph pneumotachograph system with animated incentive software (Spirotrac, Vitaograph, UK) in a dedicated research clinic by trained technicians. Calibration checks were performed with a standard 3L calibration syringe according to the manufacturer's instructions at the start of each half-day clinic session. Subjects were seated with a nose clip in place and were asked to inhale to total lung capacity (TLC), then instructed to perform a forced expiration, through a mouthpiece, to residual volume (RV). The test was repeated at intervals of 30 seconds until 3 technically acceptable traces were obtained from a maximum of eight attempts. Forced expiratory volume in one second (FEV_1_) and Forced vital capacity (FVC) were recorded and the data expressed as FEV_1_ % predicted and FEV_1_/FVC ratio.

### IOW

Pre-bronchodilator lung function tests were conducted at 10, 18, and 26 years of age. Forced vital capacity (FVC), forced expiratory volume in 1 second (FEV1) were measured using a Koko Spirometer and software with a portable desktop device (both PDS Instrumentation, Louisville, KY, USA). Spirometry was performed and evaluated according to the American Thoracic Society (ATS) criteria. The children or adults, respectively, were required to be free of respiratory infection for 2 weeks and not to be taking any oral steroids and were advised to abstain from any β-agonist medication for 6 h and from caffeine intake for at least 4 h prior to lung function assessment.

## Definitions of variables (demographic, exposures and outcomes)

Postal questionnaires were used in ALSPAC, while interviewer-administered questionnaires were employed in MAAS and IOW available on multiple occasions from infancy to adolescence.

Wheezing reports were available at 14 time points in ALSPAC over 16.5 years, 7 in MAAS over 20 years, and 6 in IOW over 26 years.

*Parental history of asthma*, eczema and hay fever were assessed by questionnaires and were defined based on the responses given to the question "have you (and/or your partner) ever had asthma/eczema/hay fever".

*Maternal and paternal smoking* were defined based on the response given to the question "do you (or does your partner) smoke", administered during pregnancy or 1^st^ year of study child.

*Low birth weight* was defined as birth weight less than 2500 g based on NHS birth records.

*Current wheeze:* Current wheeze was defined as a positive response to either the question "Has your child had wheezing in the last 12 months" or "Has your child had wheezing with whistling in the last 12 months" in ALSPAC, and in the other three cohorts to the question "Has your child had wheezing or whistling in the chest in the last 12 months?".

*Current asthma*^11^*:* Presence of any two of the following three features: 1) Current wheeze; 2) Current use of asthma medication; 3) Physician-diagnosed asthma ever.

*BMI (kg/m^2^):* weight and height were measured at annual clinic visits

*Underweight and obese:* Defined using BMI z-score based on the British 1990 Growth Reference (underweight: z-score < -1, normal: >= -1 and <=1, overweight: z-score > 1 and < 2, obese: z-score > 2).

*Skin prick test (SPT):* The atopic status of the children was determined (at an annual clinic when the children were 7–8 years of age in ALSPAC^12^, at ages 4-5, and 7-8 years in MAAS and IOW) by skin prick tests to a panel of up to 12 common allergens including house dust mite, mixed grasses and cat. Sensitization to one of these three allergens has been shown to identify 95% of all sensitized children in this population. A positive response was defined as a mean weal diameter of >3 mm (>2 mm for ALSPAC) with an absent response to negative control solution, and atopy was defined as a positive response to one or more of house dust mite, cat or grass pollen.

## Data-driven phenotype allocation

Cluster allocation of our study participants into latent wheeze phenotypes has previously been performed using machine learning methods for latent variable analysis.

*Spell-based wheeze phenotypes^13^:* Based on prospectively collected current wheeze data from five pooled birth cohorts' birth to age 16 years, children were assigned as:

1. Never wheezing no or low prevalence of wheeze throughout observation period.
2. Early transient wheeze: high prevalence of wheeze during infancy, with decrease to mid-childhood.
3. Intermittent wheeze: wheeze from infancy to adolescence interspersed with periods of no wheeze.
4. Late-onset wheezing: low prevalence until mid-childhood age, increasing rapidly to a peak prevalence in adolescence.
5. Persistent wheeze*:* high prevalence of wheeze throughout.

# STATISTICAL ANALYSIS

## Definition and derivation of spirometry phenotypes: Sensitivity analyses

Using pooled data, we performed regression analysis and estimated the regression residuals for FVC and FEV1/FVC adjusted for age, height, and race/ethnicity after stratification by sex. The residual for each participant is the difference between his/her actual spirometry and expected spirometry. The assignments to spirometry phenotypes (normal, restrictive, obstructive) were further tested in two sensitivity analyses. The first used regression residuals, with age and height as quadratic terms in the model, and the second added cohort as a fixed effect.

We used regression residuals rather than GLI for the following reasons:

1. To ensure that our finding are comparable with previous studies; We adopted the exact definition used in a recent study by Voraphani et al. (Lancet Respir Med 2022; 10(1): 59-71), which reported both spirometric obstruction and restriction in early adulthood and the associated risk factors

2. A study by Wang et al. which used data from 14 population-based cohorts (and to which we contributed) reported a high heterogeneity in GLI fit between age groups and cohorts (ERJ Open Res. 2021 Dec 6;7(4):00457-2021.). GLI fit estimates for FEV1, FVC and FEV1/ FVC z-scores were outside the suggested range for six, four and three cohorts, respectively.

Finally, Figure S10 show that the regression residuals are more consistent across our three cohorts than the GLI Z-score, particularly at age 15-18.

## Descriptive analyses

To look at the transition rates of spirometry phenotypes, we calculated all possible transitions from mid-school age to adolescence, and from adolescence to early adulthood. To investigate risk profiles of these transitions, we associated these with both early life and mid-schools risk factors.

## Data driven analysis: Patterns of change in spirometry phenotypes over time

We first derived sequences of spirometry phenotypes over time. We then used Hierarchical Clustering coupled with optimal matching to derive clusters of the sequences. We used the Elbow and silhouette method to select optimal number of clusters.

## Hierarchical clustering:

 Hierarchical clustering also known as hierarchical cluster analysis, is an algorithm that groups similar objects into groups called clusters. The endpoint is a set of clusters, where each cluster is distinct from each other cluster, and the objects within each cluster are broadly similar to each other.

## Optimal Matching:

Optimal matching distance measures pairwise dissimilarities between sequences, and then identifies ‘types’ of patterns by clustering the sequences based on these dissimilarities. OM measures the dissimilarity between two sequences, x and y, as the minimum total cost of transforming one sequence, say x, into the other sequence, y, by means of indels—either inserts or deletes—of tokens or substitutions between tokens. Each operation is assigned a cost, which may vary with the involved states^14^.

To investigate how sensitive our results are to missing data, we repeated the analysis in participants having lung function data at all three time points and spirometry phenotypes imputed data.

## Missing data and imputation

Missing data was observed both in spirometry and risk factors. The missing data pattern and percentages are given in Figure S10. Overall, 10.7% percent of data was missing, whereas variables-specific data ranges from 0% to 33%. To impute missing data, we used multiple imputations (MI); MI assumes that the data are missing at random (MAR). Although the MAR assumption cannot be verified with the data and it can be questionable in some situations, the assumption becomes more plausible as more variables are included in the imputation model^15^.

We used SAS PROC MI to impute risk factors and spirometry data using multivariate normal distribution and fully conditional specification (also known as imputation by chained equations/ICE or sequential generalized regression)^16^, where the results were comparable between both methods. The multiply imputed data were analyzed using PROC MIANALYZE.

**Figure S1:** Missing data patterns for data driven analysis (N=6103)

**
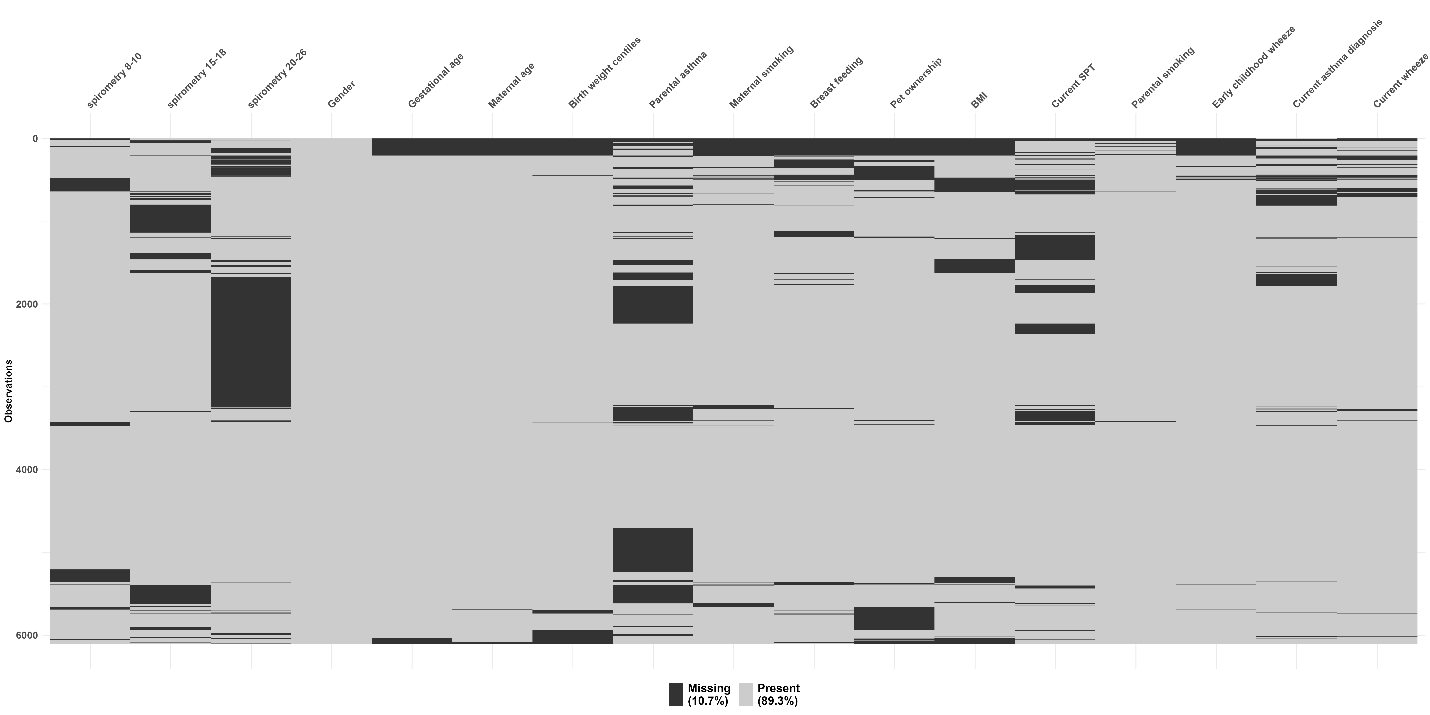
**

**
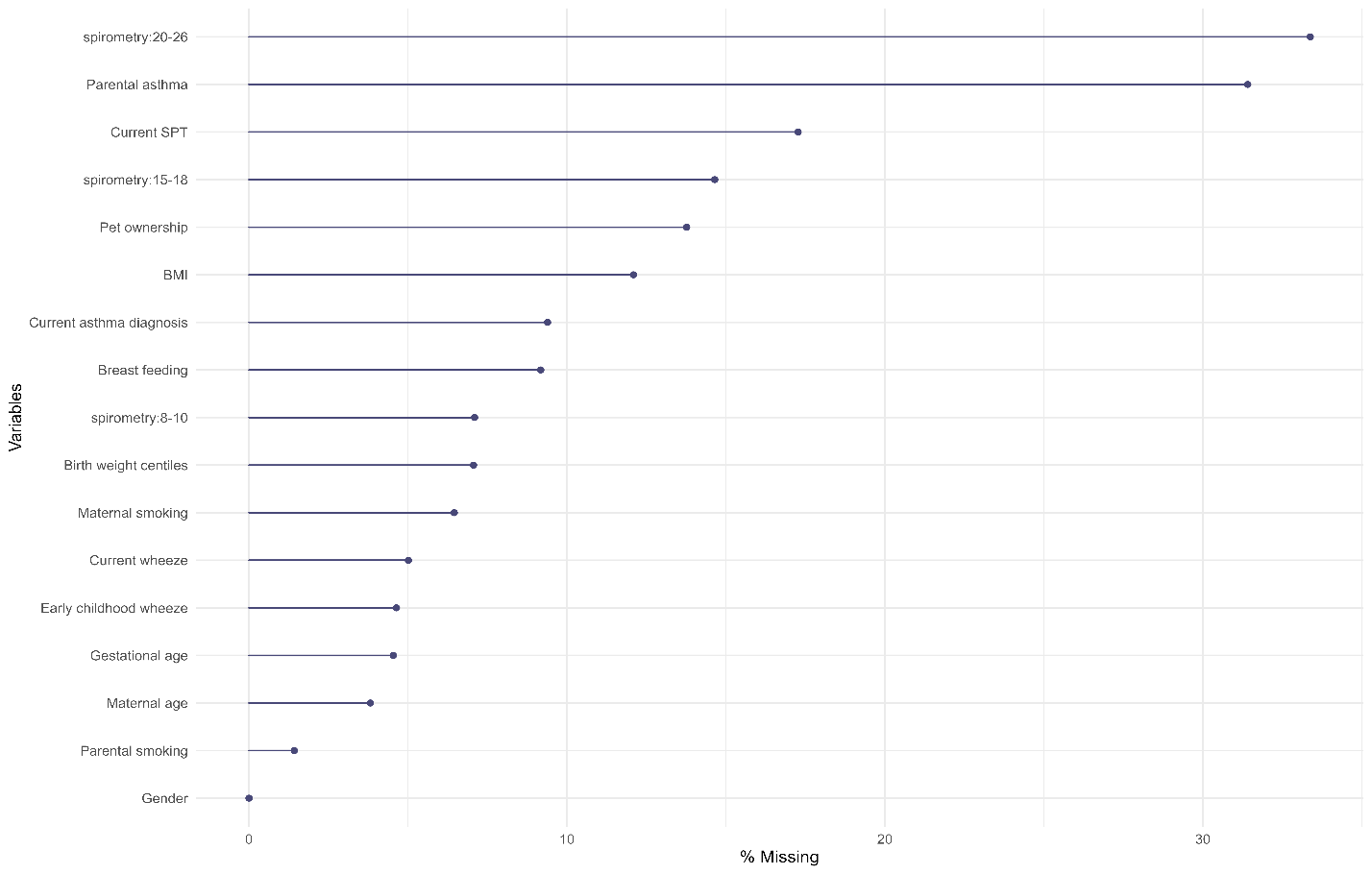
**

## The list of variables included in the analyses of the associates of spirometry clusters

We included the following variables in the analysis, these variables were selected *a priori* using literature. We only included risk factors that are available across all cohorts and could be harmonized:

*Demographic and early life:*

1. Gestational age (weeks)
2. Maternal age (years)
3. Maternal smoking during pregnancy
4. Parental asthma (current and ever)
5. Parental smoking at the time of first lung function
6. Birth weight (centiles)
7. Breastfeeding
8. Early childhood wheeze (current wheeze at follow-up up to age 5 years).

*At the time of first lung function:*

1. BMI for age z-score
2. Current wheeze
3. Current asthma diagnosis
4. Allergic sensitization.

Gestational age-adjusted birth weight (z-scores and centiles) was calculated using the tools developed by the International Fetal and Newborn Growth Consortium for the 21^st^ Century.^17^ BMI for age z-scores and BMI for age categories (underweight: z-score<-1; normal: z-score>-1 and <1; overweight: z-score >1 and <2, obese: z-score>2) at the time of first lung function assessment were calculated based on the British 1990 Growth Reference.^18^

# SUPPLEMENTARY RESULTS

## DEMOGRAPHICS, PREVALENCE OF SPIROMETRY PHENOTYPES, AND THEIR ASSOCIATES

**Table S1:** Sample sizes at each age by cohort

| Cohort | **8-10 Year** | **15-18 Year** | **20-26 Year** |
| --- | --- | --- | --- |
| ALSPAC | 6646 | 4362 | 3642 |
| IOW | 980 | 837 | 546 |
| MAAS | 778 | 565 | 492 |
| **Pooled** | **8404** | **5764** | **4680** |

**Table S2:** Characteristics of the study populations: ALSPAC, IOW, and MAAS.

|  | **ALSPAC** | **IOW** | **MAAS** |
| --- | --- | --- | --- |
| Male | 3344/6646 (50.32%) | 488/980 (49.80%) | 412/778 (52.96%) |
| Gestational age (weeks) | 39.46 ± 1.86 | 39.92 ± 1.54 | 39.93 ± 1.57 |
| Maternal age (years) | 29.12 ± 4.56 | 26.97 ± 5.24 | 30.69 ± 4.80 |
| Birth weight centiles | 63.32 ± 27.89 | 56.80 ± 29.39 | 61.72 ± 28.46 |
| Parental asthma | 667/3997 (16.69%) | 194/976 (19.88%) | 229/778 (29.43%) |
| Maternal smoking during pregnancy | 1245/6072 (20.50%) | 217/969 (22.39%) | 98/775 (2.65%) |
| Breast feeding first six months | 4799/5882 (81.59%) | 731/912 (80.15%) | 548/740 (74.05%) |
| Early childhood wheeze^a^ | 3083/6239 (49.41%) | 276/957 (28.84%) | 341/778 (43.83%) |
| Pet ownership in the first year of life^b^ | 4082/5920 (68.95%) | 504/919 (54.84%) | 130/348 (37.36%) |
| 1. current wheeze at follow-up up to age 5 years. 2. Pet inside the house or contact with pets most of the time in first year of life. | | | |

**Figure S2:** Overall (a) and cohort-specific prevalence of spirometry phenotypes over time:

b) ALSPAC;

c) IoW;

d) MAAS


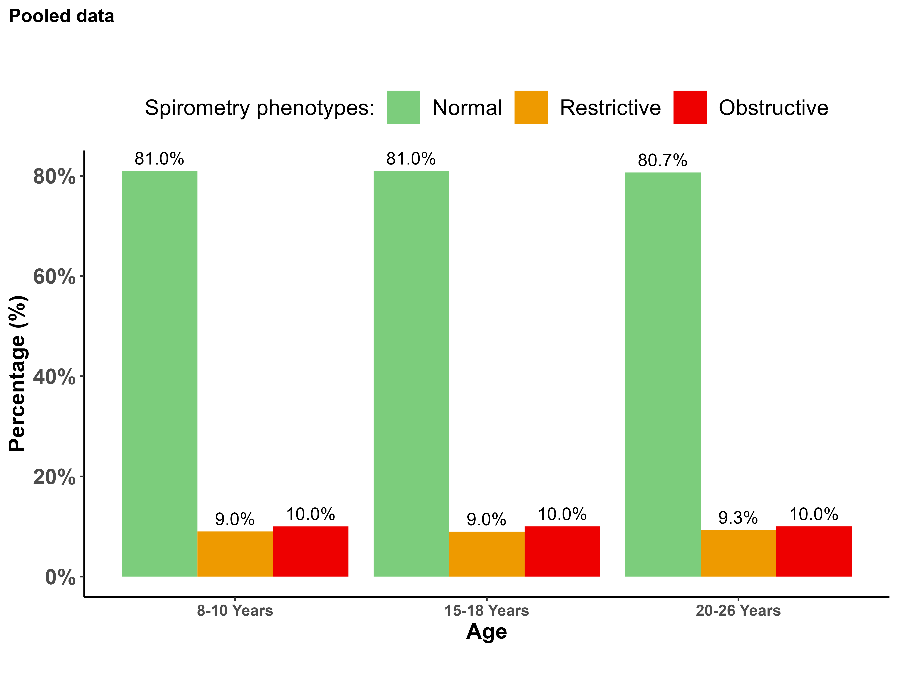


**a)**

**d)**

**c)**

**c)**

**b)**

**b)**


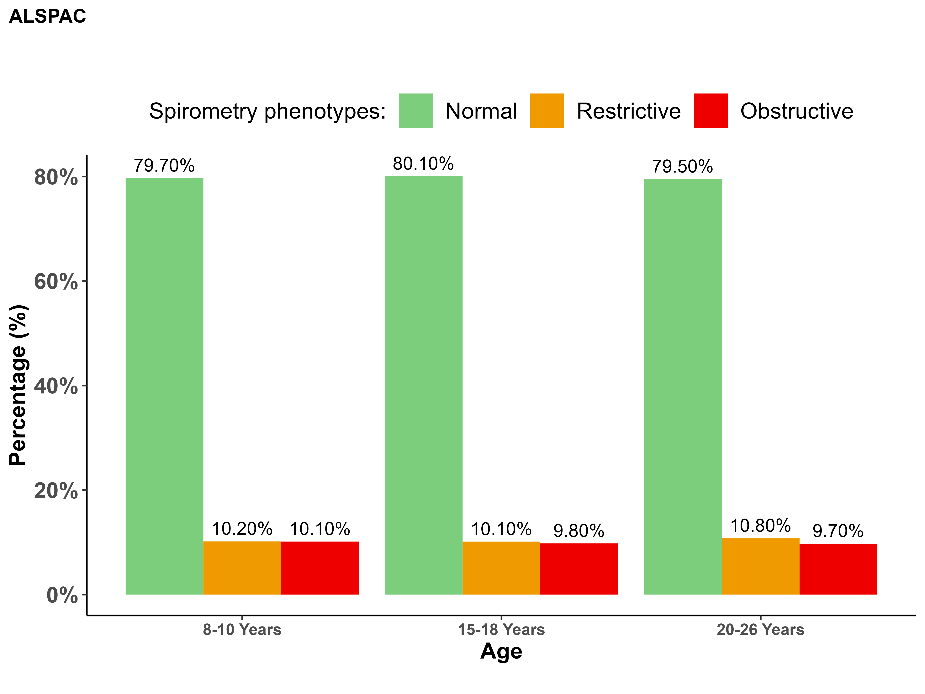

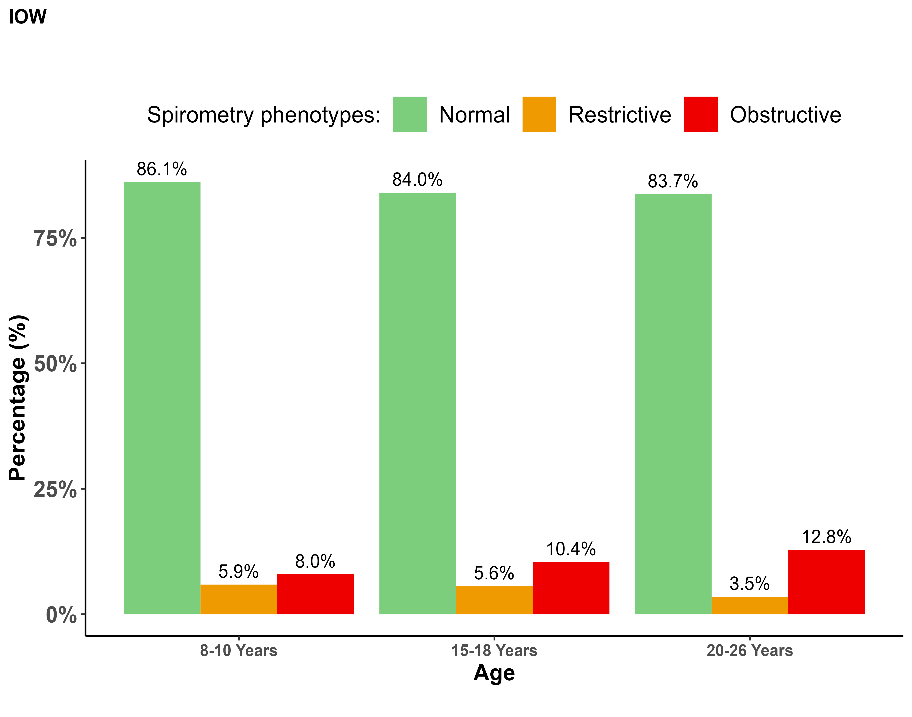

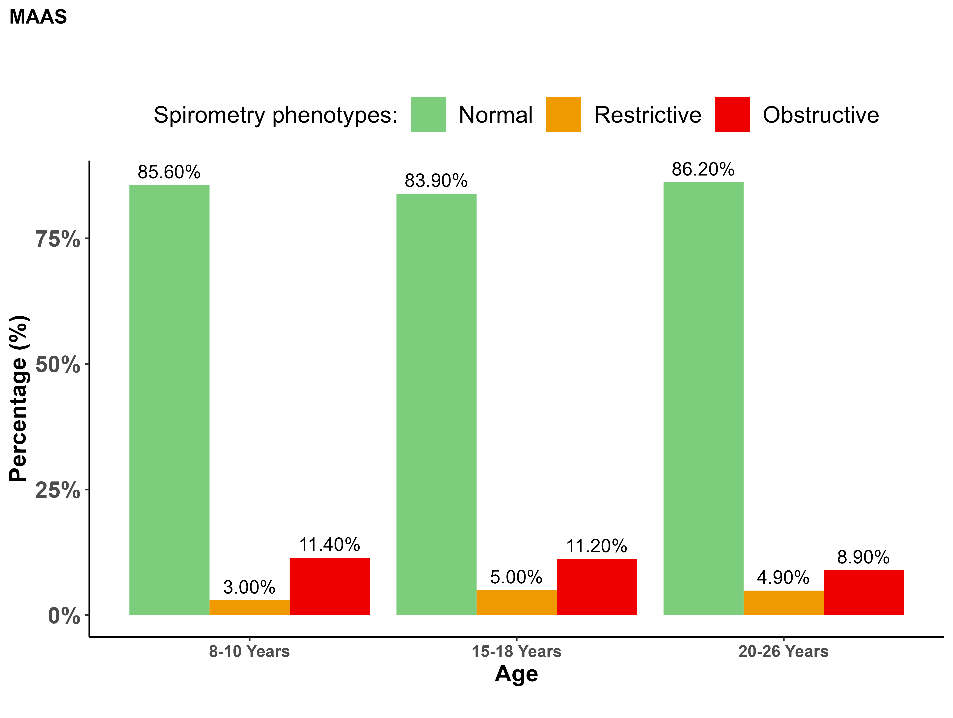


**Table S3:** Mean and standard deviations of FVC and FEV_1_/FVC percent predicted (GLI) by observed spirometry pattern (pooled data).

|  | **Normal** | **Restrictive** | **Obstructive** | **P-value** |
| --- | --- | --- | --- | --- |
| **8-10 Years** |  |  |  |  |
| FVC % predicted | 101.70 ± 9.99 | 78.71 ± 5.66 | 104.60 ± 12.79 | <.001 |
| FEV_1_ % predicted | 103.0 ± 10.15 | 83.26 ± 6.87 | 90.25 ± 11.51 | <.001 |
| FEV_1_/FVC % predicted | 100.60 ± 5.28 | 105.0 ± 5.73 | 85.71 ± 4.81 | <.001 |
| **15-18 Years** |  |  |  |  |
| FVC % predicted | 93.73 ± 11.89 | 66.26 ± 9.21 | 96.24 ± 15.56 | <.001 |
| FEV_1_ % predicted | 97.14 ± 12.15 | 70.71 ± 10.45 | 82.25 ± 15.46 | <.001 |
| FEV_1_/FVC % predicted | 103.20 ± 6.11 | 105.80 ± 6.13 | 84.80 ± 7.57 | <.001 |
| **20-26 Years** |  |  |  |  |
| FVC % predicted | 99.93 ± 9.58 | 77.36 ± 6.56 | 103.40 ± 14.07 | <.001 |
| FEV_1_ % predicted | 98.51 ± 9.62 | 78.75 ± 8.01 | 84.98 ± 12.19 | <.001 |
| FEV_1_/FVC % predicted | 98.03 ± 5.76 | 101.10 ± 6.87 | 81.69 ± 5.78 | <.001 |

**Table S4:** Univariate risk factors of spirometry phenotypes

LF: lung function; a. Pet inside the house or contact with pets most of the time in first year of life; b. Current wheeze at age five or below years.; c. Based on the British 1990 Growth Reference; d. Defined using BMI z-score based on the British 1990 Growth Reference (underweight: z-score < -1, normal: >= -1 and <=1, overweight: z-score > 1 and < 2, obese: z-score > 2).

**A: age 8-10 years (pooled data)**

|  | **Normal** | **Restrictive** | **Obstructive** | **P-value (FDR)** |
| --- | --- | --- | --- | --- |
| Male | 3432/6806 (50.43%) | 387/757 (51.12%) | 425/841 (50.54%) | 0.936  (0.936) |
| Gestational age | 39.58 ± 1.77 | 39.46 ± 1.85 | 39.41 ± 2.09 | **0.011**  **(0.016)** |
| Maternal age | 29.04 ± 4.78 | 28.59 ± 4.57 | 29.00 ± 4.63 | 0.055  (0.069) |
| Birth weight centiles | 63.21 ± 27.96 | 59.04 ± 28.75 | 60.56 ± 28.80 | **<.001**  **(<0.001)** |
| Parental asthma | 897/4708 (19.05%) | 81/477 (16.98%) | 112/566 (19.79%) | 0.474  (0.507) |
| Maternal smoking during pregnancy | 1238/6361 (19.46%) | 137/682 (20.09%) | 185/773 (23.93%) | **0.013**  **(0.018)** |
| Breastfeeding during the first six months | 4934/6133 (80.45%) | 545/660 (82.58%) | 599/741 (80.84%) | 0.419  (0.483) |
| Pet ownership first year of life^a^ | 3767/5819 (64.74%) | 458/656 (69.82%) | 491/712 (68.96%) | **0.005**  **(0.008)** |
| Early childhood wheeze^b^ | 2838/6478 (43.81%) | 342/700 (48.86%) | 520/796 (65.33%) | **<.001**  **(<0.001)** |
| BMI z-score at first LF^c^ | 0.41 ± 1.00 | 0.17 ± 1.09 | 0.52 ± 1.05 | **<.001**  **(<0.001)** |
| Current wheeze at first LF | 951/6410 (14.84%) | 101/690 (14.64%) | 261/775 (33.68%) | **<.001**  **(<0.001)** |
| Current asthma diagnosis at first LF | 1033/6069 (17.02%) | 108/628 (17.20%) | 261/712 (36.66%) | **<.001**  **(<0.001)** |
| Current sensitization at first LF | 1020/5606 (18.19%) | 100/586 (17.06%) | 189/674 (28.04%) | **<.001**  **(<0.001)** |
| Current Parental smoking at first LF | 1764/6730 (26.21%) | 158/746 (21.18%) | 247/830 (29.76%) | **<.001**  **(<0.001)** |
| BMI categories at first LF |  |  |  | **<.001**  **(<0.001)** |
| Underweight^d^ | 238/6512 (3.65%) | 63/718 (8.77%) | 26/808 (3.22%) |  |
| Normal^d^ | 4999/6512 (76.77%) | 539/718 (75.07%) | 582/808 (72.03%) |  |
| Overweight^d^ | 1002/6512 (15.39%) | 85/718 (11.84%) | 153/808 (18.94%) |  |
| Obese^d^ | 273/6512 (4.19%) | 31/718 (4.32%) | 47/808 (5.82%) |  |

**B:** Age 15-18 years (pooled data)

|  | **Normal** | **Restrictive** | **Obstructive** | P-value  **(FDR)** |
| --- | --- | --- | --- | --- |
| Male | 2241/4670 (47.99%) | 251/517 (48.55%) | 277/577 (48.01%) | 0.971  (0.971) |
| Gestational age | 39.63 ± 1.72 | 39.49 ± 1.74 | 39.43 ± 1.96 | **0.013**  **(0.024)** |
| Maternal age | 29.02 ± 4.76 | 29.28 ± 4.87 | 29.06 ± 4.84 | 0.537  (0.620) |
| Birth weight centiles | 62.54 ± 28.51 | 62.35 ± 26.97 | 60.67 ± 29.06 | 0.358  (0.488) |
| Parental asthma | 628/3243 (19.36%) | 56/338 (16.57%) | 90/390 (23.08%) | 0.079  (0.132) |
| Maternal smoking during pregnancy | 761/4350 (17.49%) | 63/470 (13.40%) | 133/545 (24.40%) | **<.001**  **(<0.001)** |
| Breastfeeding during the first six months | 3484/4202 (82.91%) | 380/459 (82.79%) | 427/519 (82.27%) | 0.935  (0.971) |
| Pet ownership first year of life^a^ | 2590/3981 (65.06%) | 301/446 (67.49%) | 312/493 (63.29%) | 0.399  (0.499) |
| Early childhood wheeze^b^ | 1924/4429 (43.44%) | 229/485 (47.22%) | 327/555 (58.92%) | **<.001**  **(<0.001)** |
| BMI z-score at first LF^c^ | 0.40 ± 0.99 | 0.07 ± 1.04 | 0.58 ± 1.03 | **<.001**  **(<0.001)** |
| Current wheeze at first LF | 666/4346 (15.32%) | 80/479 (16.70%) | 145/539 (26.90%) | **<.001**  **(<0.001)** |
| Current asthma diagnosis at first LF | 701/4160 (16.85%) | 83/449 (18.49%) | 172/517 (33.27%) | **<.001**  **(<0.001)** |
| Current sensitization at first LF | 707/3734 (18.93%) | 79/402 (19.65%) | 128/463 (27.65%) | **<.001**  **(<0.001)** |
| Current Parental smoking at first LF | 1130/4576 (24.69%) | 113/502 (22.51%) | 156/569 (27.42%) | 0.171  (0.257) |
| BMI categories at first LF |  |  |  | **<.001**  **(<0.001)** |
| Underweight^d^ | 153/3997 ( 3.83%) | 35/424 (8.25%) | 9/497 (1.81%) |  |
| Normal^d^ | 3058/3997 (76.51%) | 329/424 (77.59%) | 354/497 (71.23%) |  |
| Overweight^d^ | 626/3997 (15.66%) | 46/424 (10.85%) | 100/497 (20.12%) |  |
| Obese^d^ | 160/3997 (4.00%) | 14/424 (3.30%) | 34/497 (6.84%) |  |

**C: Age 20-26 years (pooled data).**

|  | **Normal** | **Restrictive** | **Obstructive** | **P-value**  **(FDR)** |
| --- | --- | --- | --- | --- |
| Male | 1506/3776 (39.88%) | 175/435 (40.23%) | 185/469 (39.45%) | 0.971  (0.971) |
| Gestational age | 39.61 ± 1.72 | 39.49 ± 1.84 | 39.45 ± 1.96 | 0.085  (0.128) |
| Maternal age | 29.32 ± 4.73 | 29.17 ± 4.53 | 29.36 ± 4.99 | 0.816  (0.875) |
| Birth weight centiles | 62.75 ± 28.18 | 59.09 ± 27.60 | 61.18 ± 28.84 | **0.039**  (0.072) |
| Parental asthma | 482/2580 (18.68%) | 50/274 (18.25%) | 71/327 (21.71%) | 0.400  (0.532) |
| Maternal smoking during pregnancy | 563/3452 (16.31%) | 45/375 (12.00%) | 101/433 (23.33%) | **<.001** |
| Breastfeeding in the first six months | 2801/3340 (83.86%) | 301/368 (81.79%) | 357/419 (85.20%) | 0.426  (0.532) |
| Pet ownership first year of life | 1994/3133 (63.65%) | 235/354 (66.38%) | 264/404 (65.35%) | 0.508  (0.586) |
| Early childhood wheeze | 1452/3510 (41.37%) | 165/388 (42.53%) | 255/443 (57.56%) | **<.001**  **(<0.001)** |
| BMI z-score at first LF | 0.39 ± 0.99 | 0.11 ± 1.09 | 0.44 ± 1.09 | **<.001**  **(<0.001)** |
| Current wheeze at first LF | 510/3372 (15.12%) | 52/383 (13.58%) | 114/417 (27.34%) | **<.001**  **(<0.001)** |
| Current asthma diagnosis at first LF | 548/3259 (16.81%) | 51/363 (14.05%) | 124/402 (30.85%) | **<.001**  **(<0.001)** |
| Current sensitization at first LF | 555/2872 (19.32%) | 61/320 (19.06%) | 86/352 (24.43%) | 0.072  (0.119) |
| Current Parental smoking at first LF | 857/3631 (23.60%) | 80/416 (19.23%) | 134/455 (29.45%) | **0.002**  **(0.003)** |
| BMI categories at first LF |  |  |  | **<.001**  **(<0.001)** |
| Underweight | 107/3031 (3.53%) | 31/323 (9.60%) | 15/368 (4.08%) |  |
| Normal | 2303/3031 (75.98%) | 239/323 (73.99%) | 266/368 (72.28%) |  |
| Overweight | 498/3031 (16.43%) | 44/323 (13.62%) | 63/368 (17.12%) |  |
| Obese | 123/3031 (4.06%) | 9/323 (2.79%) | 24/368 (6.52%) |  |

## CHANGE IN SPIROMETRY PHENOTYPES OVER TIME AND THE ASSOCIATES OF CHANGE

**Figure S3:** Alluvial plots for transitions among different observed spirometry patterns in each cohort.


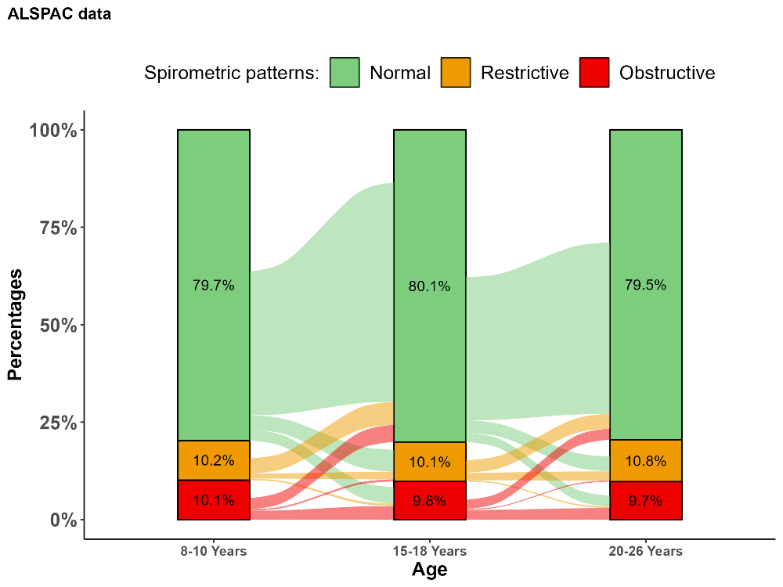


**
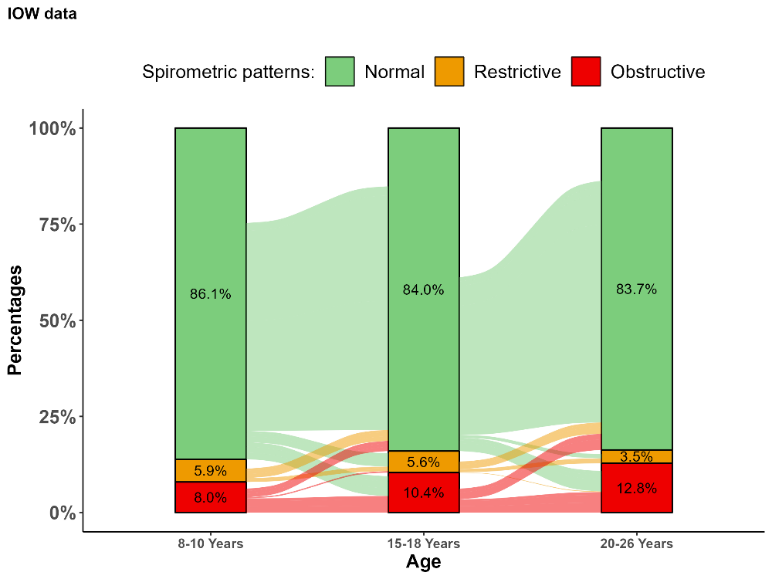
**


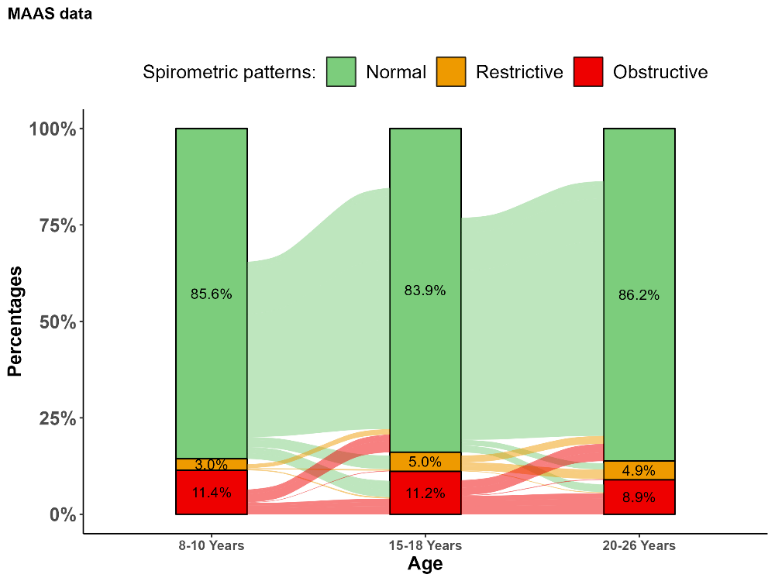


## IMPROVEMENT AND DECLINE IN SPIROMETRY PHENOTYPES OVER TIME

**Table S5:** Time-specific obstructive phenotypes (pooled data)

|  | **Time 1 to Time 2**  **8-10 years to 15-18 years** | | **Time 2 to Time 3**  **15-18 years to 20-26 years** | |
| --- | --- | --- | --- | --- |
| **Obstructive phenotypes** | **Frequency** | **Percent** | **Frequency** | **Percent** |
| **Non-Obstructive** | 3336 | 82.62 | 2268 | 83.29 |
| **Worsening** | 251 | 6.22 | 142 | 5.21 |
| **Improvement** | 240 | 5.94 | 149 | 5.47 |
| **Persistent obstruction** | 211 | 5.23 | 164 | 6.02 |

**Table S6:** Univariate risk factors of obstructive phenotypes change

**A.** First transition: 8-10 years to 15-18 years

|  | **Normal**  **(Normal to Normal)** | **Worsening**  **(Normal to Obstructive)** | **Improvement**  **(Obstructive to Normal)** | **Persistent Obstruction**  **(Obstructive to obstructive)** | **P-value**  **(FDR)** |
| --- | --- | --- | --- | --- | --- |
| Male | 1591/3336 (47.69%) | 116/251 (46.22%) | 119/240 (49.58%) | 104/211 (49.29%) | 0.859  (0.920) |
| Gestational age | 39.67 ± 1.66 | 39.48 ± 1.89 | 39.48 ± 1.99 | 39.21 ± 2.26 | **<.001**  **(0.002)** |
| Maternal age | 29.21 ± 4.71 | 29.08 ± 5.09 | 29.05 ± 4.74 | 29.52 ± 4.40 | 0.715  (0.825) |
| Birth weight centiles | 63.15 ± 28.50 | 61.01 ± 29.48 | 61.00 ± 28.97 | 61.12 ± 28.13 | 0.398  (0.542) |
| Parental asthma | 453/2362 (19.18%) | 44/174 (25.29%) | 27/152 (17.76%) | 31/149 (20.81%) | 0.230  (0.384) |
| Maternal smoking during pregnancy | 514/3153 (16.30%) | 62/241 (25.73%) | 36/228 (15.79%) | 42/196 (21.43%) | **<.001**  **(0.002)** |
| Breastfeeding during the first six months | 2565/3076 (83.39%) | 187/221 (84.62%) | 185/217 (85.25%) | 156/195 (80.00%) | 0.493  (0.617) |
| Pet ownership first year of life^a^ | 1878/2914 (64.45%) | 136/216 (62.96%) | 138/209 (66.03%) | 117/184 (63.59%) | 0.921  (0.921) |
| Early childhood wheeze^b^ | 1328/3213 (41.33%) | 121/241 (50.21%) | 142/228 (62.28%) | 141/205 (68.78%) | **<.001**  **(<0.001)** |
| BMI z-score at first LF^c^ | 0.42 ± 0.98 | 0.59 ± 1.04 | 0.51 ± 1.03 | 0.57 ± 1.00 | **0.016**  **(0.035)** |
| Current wheeze at first LF | 464/3231 (14.36%) | 50/243 (20.58%) | 77/228 (33.77%) | 65/203 (32.02%) | **<.001**  **(<0.001)** |
| Current asthma diagnosis at first LF | 492/3078 (15.98%) | 60/234 (25.64%) | 67/211 (31.75%) | 74/192 (38.54%) | **<.001**  **(<0.001)** |
| Current sensitization at first LF | 532/2888 (18.42%) | 48/211 (22.75%) | 57/202 (28.22%) | 58/182 (31.87%) | **<.001**  **(<0.001)** |
| Current Parental smoking at first LF | 848/3305 (25.66%) | 73/250 (29.20%) | 66/238 (27.73%) | 64/209 (30.62%) | 0.256  (0.384) |
| BMI categories at first LF |  |  |  |  | **0.044**  **(0.083)** |
| Underweight^d^ | 109/3229 (3.38%) | 5/239 (2.09%) | 7/233 (3.00%) | 3/203 (1.48%) |  |
| Normal^d^ | 2477/3229 (76.71%) | 166/239 (69.46%) | 172/233 (73.82%) | 149/203 (73.40%) |  |
| Overweight^d^ | 511/3229 (15.83%) | 51/239 (21.34%) | 45/233 (19.31%) | 39/203 (19.21%) |  |
| Obese^d^ | 132/3229 (4.09%) | 17/239 ( 7.11%) | 9/233 (3.86%) | 12/203 (5.91%) |  |

**B:** Second transition: 15-18 years to 20-26 years

|  | **Normal**  **(Normal to Normal)** | **Worsening**  **(Normal to Obstructive)** | **Improvement**  **(Obstructive to Normal)** | **Persistent Obstruction**  **(Obstructive to Obstructive)** | **P-value** |
| --- | --- | --- | --- | --- | --- |
| Male | 923/2268 (40.70%) | 60/142 (42.25%) | 57/149 (38.26%) | 72/164 (43.90%) | 0.757  (0.757) |
| Gestational age | 39.67 ± 1.73 | 39.66 ± 1.62 | 39.77 ± 1.65 | 39.27 ± 2.08 | **0.038**  **(0.045)** |
| Maternal age | 29.47 ± 4.68 | 29.81 ± 5.02 | 29.15 ± 5.02 | 29.56 ± 4.73 | 0.702  (0.752) |
| Birth weight centiles | 63.05 ± 28.41 | 61.67 ± 29.78 | 61.03 ± 28.26 | 59.31 ± 28.32 | 0.385  (0.444) |
| Parental asthma | 321/1614 (19.89%) | 18/102 (17.65%) | 23/99 (23.23%) | 34/116 (29.31%) | 0.077  (0.159) |
| Maternal smoking during pregnancy | 309/2135 (14.47%) | 26/135 (19.26%) | 28/142 (19.72%) | 40/157 (25.48%) | **<.001**  **(0.003)** |
| Breast feeding first six months | 1776/2083 (85.26%) | 115/131 (87.79%) | 110/136 (80.88%) | 131/150 (87.33%) | 0.354  (0.442) |
| Pet ownership first year of life^a^ | 1206/1930 (62.49%) | 91/129 (70.54%) | 82/124 (66.13%) | 78/139 (56.12%) | 0.085  (0.159) |
| Early childhood wheeze^b^ | 874/2168 (40.31%) | 74/138 (53.62%) | 75/144 (52.08%) | 95/158 (60.13%) | **<.001**  **(<0.001)** |
| BMI z-score at first LF^c^ | 0.40 ± 0.99 | 0.36 ± 1.10 | 0.60 ± 0.96 | 0.49 ± 1.08 | 0.124  (0.187) |
| Current wheeze at first LF | 318/2159 (14.73%) | 39/133 (29.32%) | 35/143 (24.48%) | 43/159 (27.04%) | **<.001**  **(<0.001)** |
| Current asthma diagnosis at first LF | 339/2088 (16.24%) | 46/128 (35.94%) | 39/138 (28.26%) | 50/153 (32.68%) | **<.001**  **(<0.001)** |
| Current sensitization at first LF | 369/1888 (19.54%) | 30/118 (25.42%) | 33/126 (26.19%) | 37/134 (27.61%) | **0.025**  **(0.075)** |
| Current Parental smoking at first LF | 541/2231 (24.25%) | 45/139 (32.37%) | 40/147 (27.21%) | 46/161 (28.57%) | 0.104  (0.174) |
| BMI categories at first LF |  |  |  |  | 0.342  (0.442) |
| Underweight^d^ | 64/2035 (3.14%) | 6/126 (4.76%) | 2/136 (1.47%) | 3/142 (2.11%) |  |
| Normal^d^ | 1550/2035 (76.17%) | 95/126 (75.40%) | 99/136 (72.79%) | 103/142 (72.54%) |  |
| Overweight^d^ | 336/2035 (16.51%) | 17/126 (13.49%) | 28/136 (20.59%) | 25/142 (17.61%) |  |
| Obese^d^ | 85/2035 (4.18%) | 8/126 (6.35%) | 7/136 (5.15%) | 11/142 (7.75%) |  |
| LF: lung function   1. Pet inside the house or contact with pets most of the time in first year of life. 2. Current wheeze at age five or below years. 3. Based on the British 1990 Growth Reference 4. Defined using BMI z-score based on the British 1990 Growth Reference (underweight: z-score < -1,   normal: >= -1 and <=1, overweight: z-score > 1 and < 2, obese: z-score > 2). | | | | | |

**Figure S4: Risk factors:** Importance plot of 10 most important risk factors from the multinomial logistic regression with obstructive phenotypes change at first transition as the response variable. The bar length for specific risk factors represents the magnitude of the regression coefficient for that risk factor. The bar placement below or above zero represents the direction of association (positive or negative). The top variables contribute more to the model than the bottom ones and also have high predictive power.


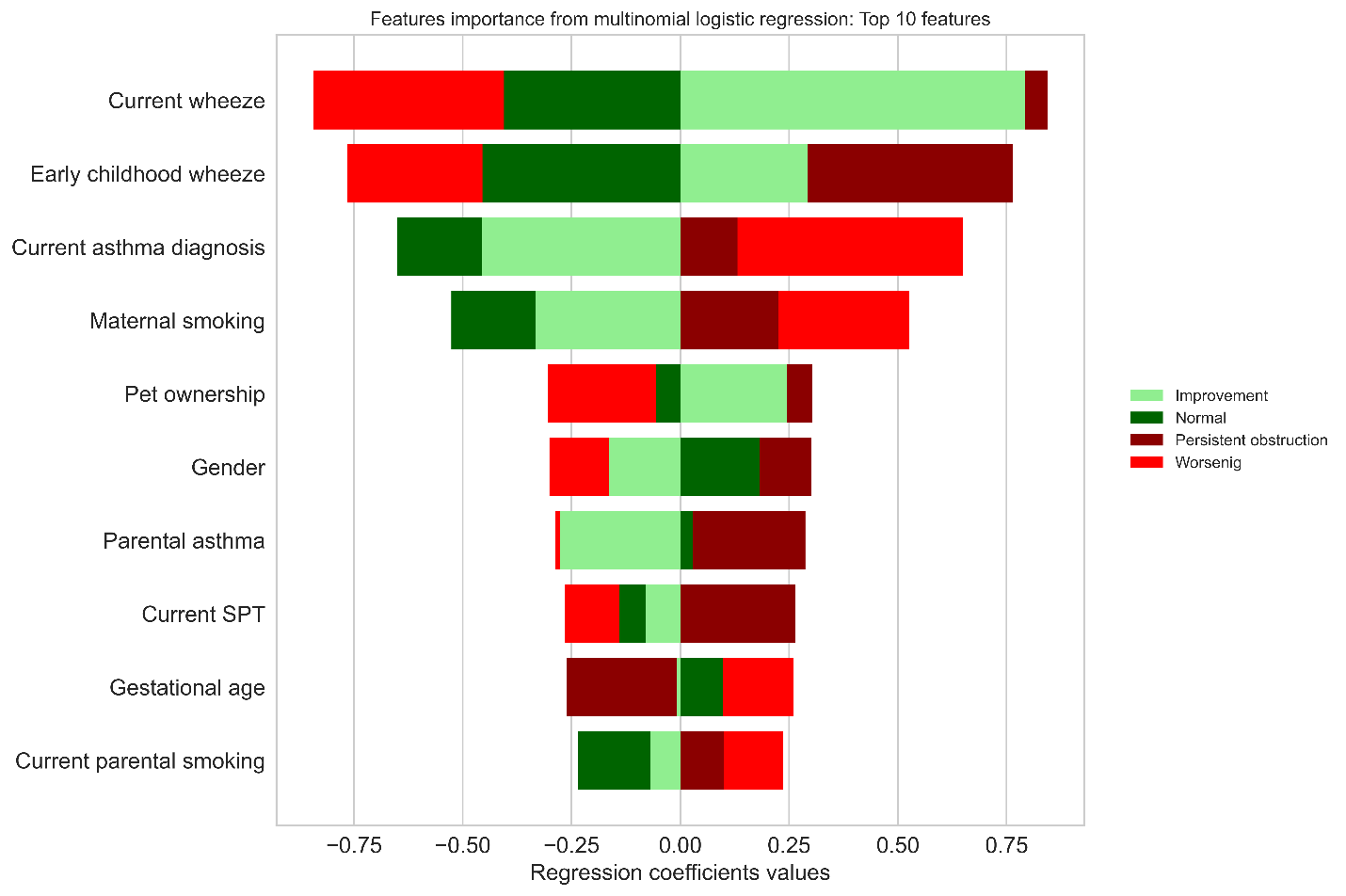


**Table S7:** Time-specific restrictive phenotypes (pooled data)

|  | **Time 1 to Time 2**  **8-10 years to 15-18 years** | | **Time 2 to Time 3**  **15-18 years to 20-26 years** | |
| --- | --- | --- | --- | --- |
| **Restrictive phenotypes** | **Frequency** | **Per cent** | **Frequency** | **Per cent** |
| **Non-Restrictive** | 3336 | 83.05 | 2268 | 84.34 |
| **Worsening** | 294 | 7.32 | 153 | 5.69 |
| **Improvement** | 290 | 7.22 | 164 | 6.1 |
| **Persistent restriction** | 97 | 2.41 | 104 | 3.87 |

**Table S8:** Univariate risk factors of restrictive phenotypes at the first transition; n/N(%), or mean ± SD.

**A. First transition: 8-10 years to 15-18 years**

|  | **Normal**  **(Normal to Normal)** | **Worsening**  **(Normal to restrictive)** | **Improvement(Restrictive to Normal)** | **Persistent Restriction**  **(Restrictive to Restrictive)** | **P-value**  **(FDR)** |
| --- | --- | --- | --- | --- | --- |
| Male | 1591/3336 (47.69%) | 149/294 (50.68%) | 151/290 (52.07%) | 46/97 (47.42%) | 0.418  (0.628) |
| Gestational age | 39.67 ± 1.66 | 39.53 ± 1.77 | 39.58 ± 1.76 | 39.40 ± 1.90 | 0.224  (0.441) |
| Maternal age | 29.21 ± 4.71 | 29.41 ± 4.87 | 29.38 ± 4.50 | 29.07 ± 4.61 | 0.835  (0.835) |
| Birth weight centiles | 63.15 ± 28.50 | 64.40 ± 25.82 | 60.08 ± 28.73 | 56.00 ± 29.47 | **0.030**  **(0.149)** |
| Parental asthma | 453/2362 (19.18%) | 33/195 (16.92%) | 35/181 (19.34%) | 6/63 (9.52%) | 0.235  (0.441) |
| Maternal smoking during pregnancy | 514/3153 (16.30%) | 34/275 (12.36%) | 43/265 (16.23%) | 12/87 (13.79%) | 0.355  (0.592) |
| Breastfeeding during the first six months | 2565/3076 (83.39%) | 226/267 (84.64%) | 226/260 (86.92%) | 70/85 (82.35%) | 0.477  (0.651) |
| Pet ownership first year of life^a^ | 1878/2914 (64.45%) | 176/255 (69.02%) | 181/259 (69.88%) | 54/85 (63.53%) | 0.174  (0.441) |
| Early childhood wheezeb | 1328/3213 (41.33%) | 126/281 (44.84%) | 134/271 (49.45%) | 35/89 (39.33%) | **0.048**  **(0.179)** |
| BMI z-score at first LF^c^ | 0.42 ± 0.98 | 0.16 ± 0.98 | 0.25 ± 1.11 | -0.27 ± 1.05 | **<.001**  **(<0.001)** |
| Current wheeze at first LF | 464/3231 (14.36%) | 46/280 (16.43%) | 40/281 (14.23%) | 11/95 (11.58%) | 0.670  (0.827) |
| Current asthma diagnosis at first LF | 492/3078 (15.98%) | 47/265 (17.74%) | 44/258 (17.05%) | 12/88 (13.64%) | 0.772  (0.827) |
| Current sensitization at first LF | 532/2888 (18.42%) | 48/245 (19.59%) | 32/241 (13.28%) | 15/83 (18.07%) | 0.227  (0.441) |
| Current Parental smoking at first LF | 848/3305 (25.66%) | 67/287 (23.34%) | 69/289 (23.88%) | 22/93 (23.66%) | 0.742  (0.827) |
| BMI categories at first LF |  |  |  |  | **<.001**  **(<0.001)** |
| Underweight^d^ | 109/3229 (3.38%) | 16/281 (5.69%) | 21/281 (7.47%) | 14/94 (14.89%) |  |
| Normal^d^ | 2477/3229 (76.71%) | 226/281 (80.43%) | 211/281 (5.09%) | 70/94 (74.47%) |  |
| Overweight^d^ | 511/3229 (15.83%) | 32/281 (11.39%) | 34/281 (12.10%) | 7/94 (7.45%) |  |
| Obese^d^ | 132/3229 (4.09%) | 7/281 (2.49%) | 15/281 (5.34%) | 3/94 (3.19%) |  |

**B. Second transition. 15-18 years to 20-26 years**

|  | **Normal**  **(Normal to Normal)** | **Worsening**  **(Normal to restrictive)** | **Improvement**  **(Restrictive to Normal)** | **Persistent Restriction**  **(Restrictive to Restrictive)** | **P-value**  **(FDR)** |
| --- | --- | --- | --- | --- | --- |
| Male | 923/2268 (40.70%) | 72/153 (47.06%) | 66/164 (40.24%) | 42/104 (40.38%) | 0.482  (0.556) |
| Gestational age | 39.67 ± 1.73 | 39.51 ± 1.76 | 39.54 ± 1.52 | 39.48 ± 1.90 | 0.416  (0.520) |
| Maternal age | 29.47 ± 4.68 | 28.80 ± 4.42 | 29.16 ± 4.56 | 30.03 ± 4.80 | 0.177  (0.443) |
| Birth weight centiles | 63.05 ± 28.41 | 59.67 ± 29.80 | 63.22 ± 27.04 | 59.15 ± 25.47 | 0.332  (0.467) |
| Parental asthma | 321/1614 (19.89%) | 18/101 (17.82%) | 14/107 (13.08%) | 10/68 (14.71%) | 0.253  (0.467) |
| Maternal smoking during pregnancy | 309/2135 (14.47%) | 14/136 (10.29%) | 18/153 (11.76%) | 10/92 (10.87%) | 0.343  (0.467) |
| Breast feeding first six months | 1776/2083 (85.26%) | 115/139 (82.73%) | 130/151 (86.09%) | 70/91 (76.92%) | 0.145  (0.443) |
| Pet ownership first year of life^a^ | 1206/1930 (62.49%) | 93/130 (71.54%) | 94/147 (63.95%) | 53/82 (64.63%) | 0.220  (0.467) |
| Early childhood wheeze^b^ | 874/2168 (40.31%) | 71/143 (49.65%) | 73/157 (46.50%) | 38/93 (40.86%) | 0.081  (0.404) |
| BMI z-score at first LF^c^ | 0.40 ± 0.99 | 0.29 ± 1.17 | 0.12 ± 0.98 | -0.06 ± 1.04 | **<.001**  **(<0.001)** |
| Current wheeze at first LF | 318/2159 (14.73%) | 17/146 (11.64%) | 30/157 (19.11%) | 14/97 (14.43%) | 0.323  (0.467) |
| Current asthma diagnosis at first LF | 339/2088 (16.24%) | 17/138 (12.32%) | 33/150 (22.00%) | 13/87 (14.94%) | 0.152  (0.443) |
| Current sensitization at first LF | 369/1888 (19.54%) | 23/121 (19.01%) | 23/127 (18.11%) | 18/85 (21.18%) | 0.954  (0.954) |
| Current Parental smoking at first LF | 541/2231 (24.25%) | 34/151 (22.52%) | 36/161 (22.36%) | 18/98 (18.37%) | 0.539  (0.578) |
| BMI categories at first LF |  |  |  |  | **0.002**  **(0.014)** |
| Underweight^d^ | 64/2035 (3.14%) | 13/131 (9.92%) | 8/138 (5.80%) | 7/88 (7.95%) |  |
| Normal^d^ | 1550/2035 (76.17%) | 92/131 (70.23%) | 111/138 (80.43%) | 68/88 (77.27%) |  |
| Overweight^d^ | 336/2035 (16.51%) | 20/131 (15.27%) | 14/138 (10.14%) | 11/88 (2.50%) |  |
| Obese^d^ | 85/2035 (4.18%) | 6/131 (4.58%) | 5/138 (3.62%) | 2/88 (2.27%) |  |
| a-d: As per definitions in Table S10 | | | | | |

**Table S9:** Frequencies and percentages of Lung function phenotypes sequences over time for participants with complete data i.e., spirometry available at all three time points (N=2739).

| **8-l0 years** | **15-18 years** | **20-26 years** | | **Frequency** | **Percent** |
| --- | --- | --- | --- | --- | --- |
| Normal | Normal | Normal | 1771 | | 79.1 |
| Normal | Restrictive | Normal | 105 | | 4.69 |
| Normal | Obstructive | Normal | 93 | | 4.16 |
| Normal | Normal | Restrictive | 86 | | 3.84 |
| Normal | Normal | Obstructive | 82 | | 3.66 |
| Normal | Obstructive | Obstructive | 48 | | 2.14 |
| Normal | Restrictive | Restrictive | 44 | | 1.97 |
| Normal | Restrictive | Obstructive | 7 | | 0.31 |
| Normal | Obstructive | Restrictive | 3 | | 0.13 |
|  |  | **Total** | **2239** | | **100** |
| Restrictive | Normal | Normal | 103 | | 47.69 |
| Restrictive | Restrictive | Restrictive | 38 | | 17.59 |
| Restrictive | Normal | Restrictive | 29 | | 13.43 |
| Restrictive | Restrictive | Normal | 18 | | 8.33 |
| Restrictive | Normal | Obstructive | 10 | | 4.63 |
| Restrictive | Obstructive | Restrictive | 7 | | 3.24 |
| Restrictive | Obstructive | Normal | 5 | | 2.31 |
| Restrictive | Restrictive | Obstructive | 3 | | 1.39 |
| Restrictive | Obstructive | Obstructive | 3 | | 1.39 |
|  |  | **Total** | **216** | | **100** |
| Obstructive | Normal | Normal | 95 | | 33.45 |
| Obstructive | Obstructive | Obstructive | 89 | | 31.34 |
| Obstructive | Normal | Obstructive | 36 | | 12.67 |
| Obstructive | Obstructive | Normal | 33 | | 11.62 |
| Obstructive | Restrictive | Normal | 12 | | 4.23 |
| Obstructive | Normal | Restrictive | 8 | | 2.82 |
| Obstructive | Restrictive | Obstructive | 6 | | 2.11 |
| Obstructive | Restrictive | Restrictive | 4 | | 1.41 |
| Obstructive | Obstructive | Restrictive | 1 | | 0.35 |
|  |  | **Total** | **284** | | **100** |

## DATA-DRIVEN ANALYSES TO IDENTIFY CLUSTERS OF THE SPIROMETRY SEQUENCES OVER TIME

**Table S10:** Characteristics of the included and excluded study populations in cluster analysis.

|  | **Included: Subjects with data on lung function at 2-3 points** | **Excluded: Subjects with data on lung function at < 2-time points** |
| --- | --- | --- |
| Male | 2828/6103 (46.34%) | 2092/3902 (53.61%) |
| Gestational age | 29.23 ± 4.71 | 28.30 ± 4.89 |
| Maternal age | 39.59 ± 1.75 | 39.49 ± 1.91 |
| Birth weight centiles | 62.35 ± 28.30 | 62.68 ± 27.72 |
| Parental asthma | 798/4186 (19.06%) | 487/2596 (18.76%) |
| Maternal smoking during pregnancy | 970/5709 (16.99%) | 924/3430 (26.94%) |
| BMI z-score at first LF | 0.39 ± 1.01 | 0.41 ± 1.04 |
| Current Parental smoking at first LF | 1523/6016 (25.32%) | 902/3708 (24.33%) |
| Breast feeding first six months | 4624/5543 (83.42%) | 2400/3213 (74.70%) |
| Pet ownership first year of life | 3420/5263 (64.98%) | 2060/3084 (66.80%) |
| Early childhood wheeze | 2610/5820 (44.85%) | 1692/3506 (48.26%) |

**Figure S5:** Elbow (A) and Silhouette (B) plot for hierarchical clustering (HC).

(A)

**
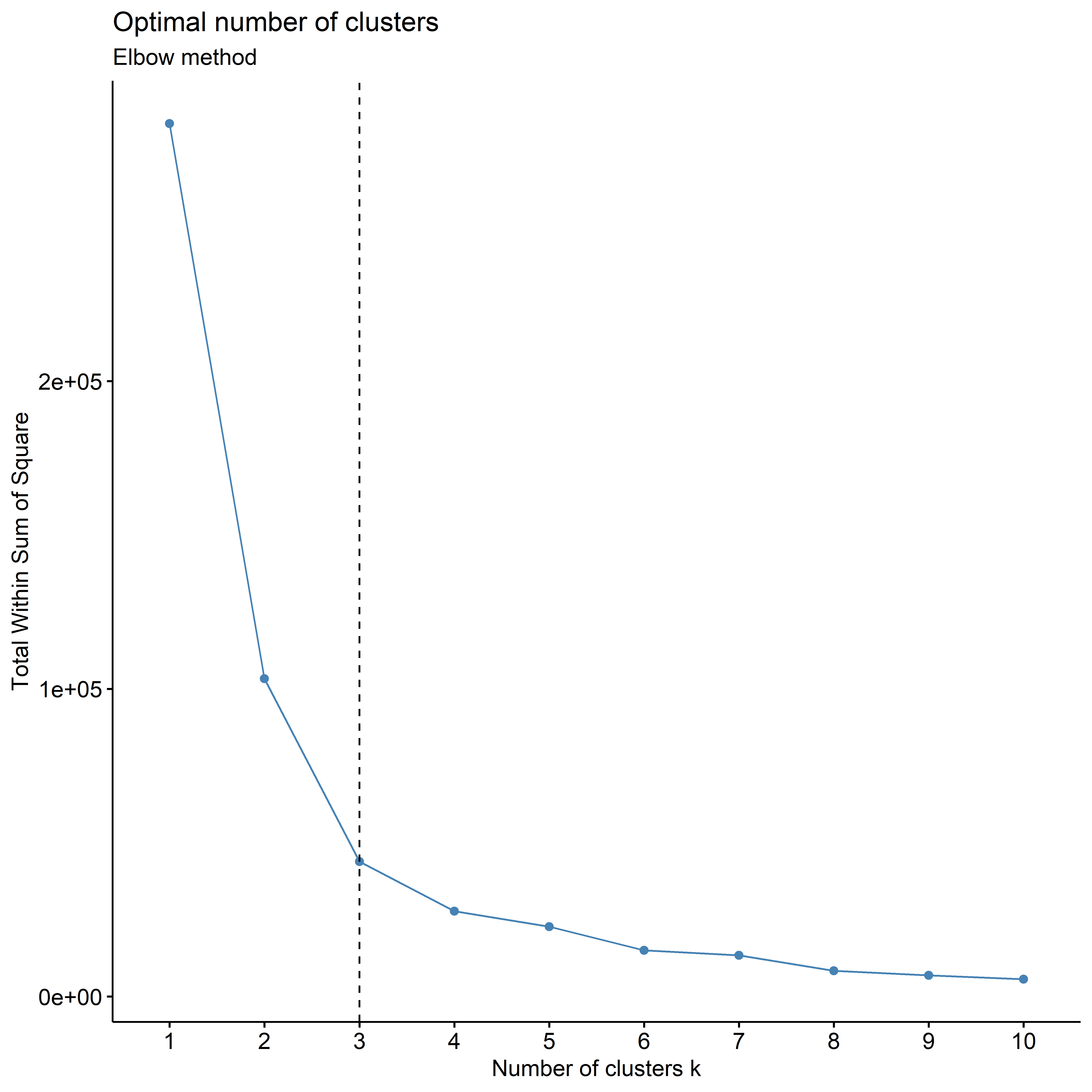
**


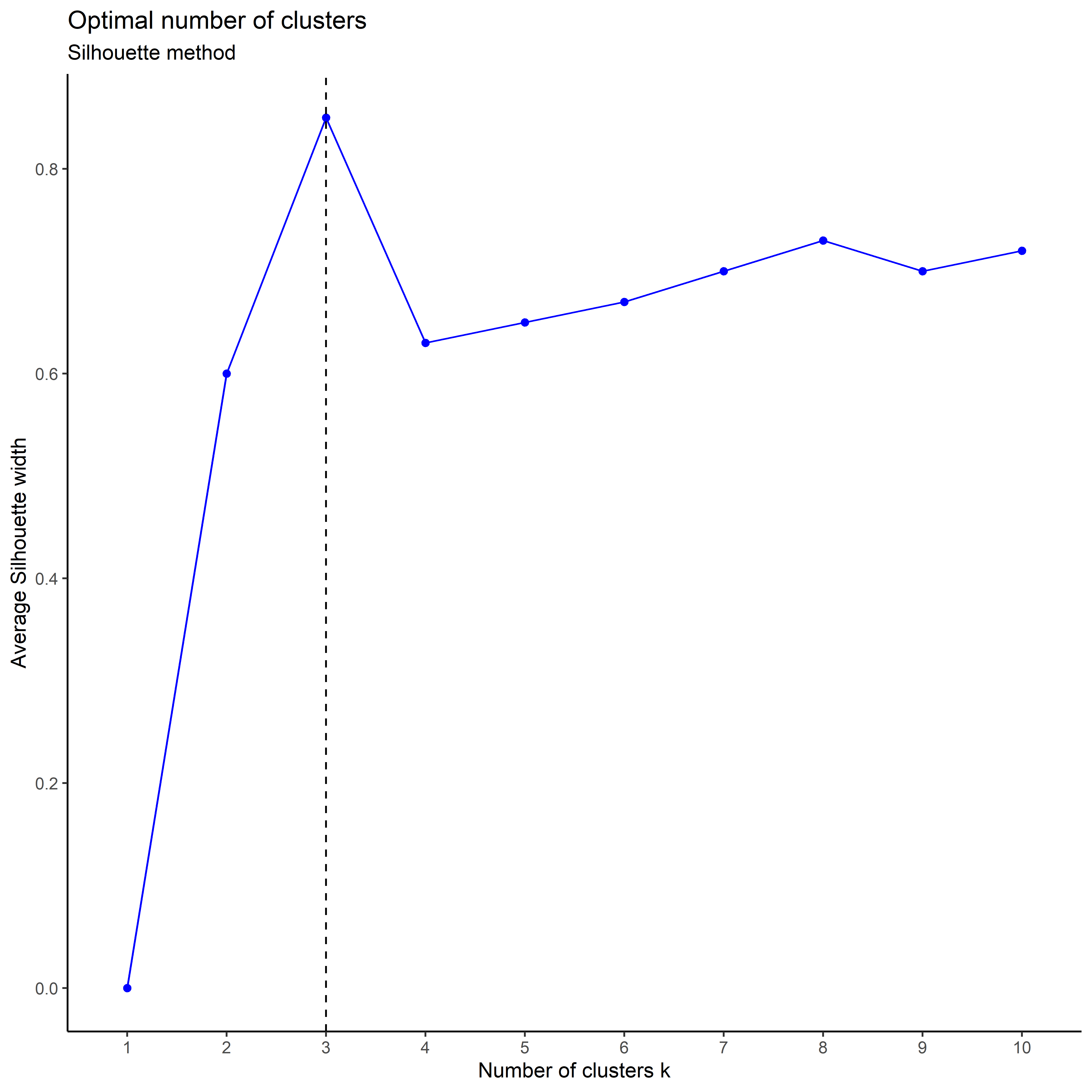
(B)

**Figure S6:** Clusters of the spirometry sequences A) complete data B) imputed data.


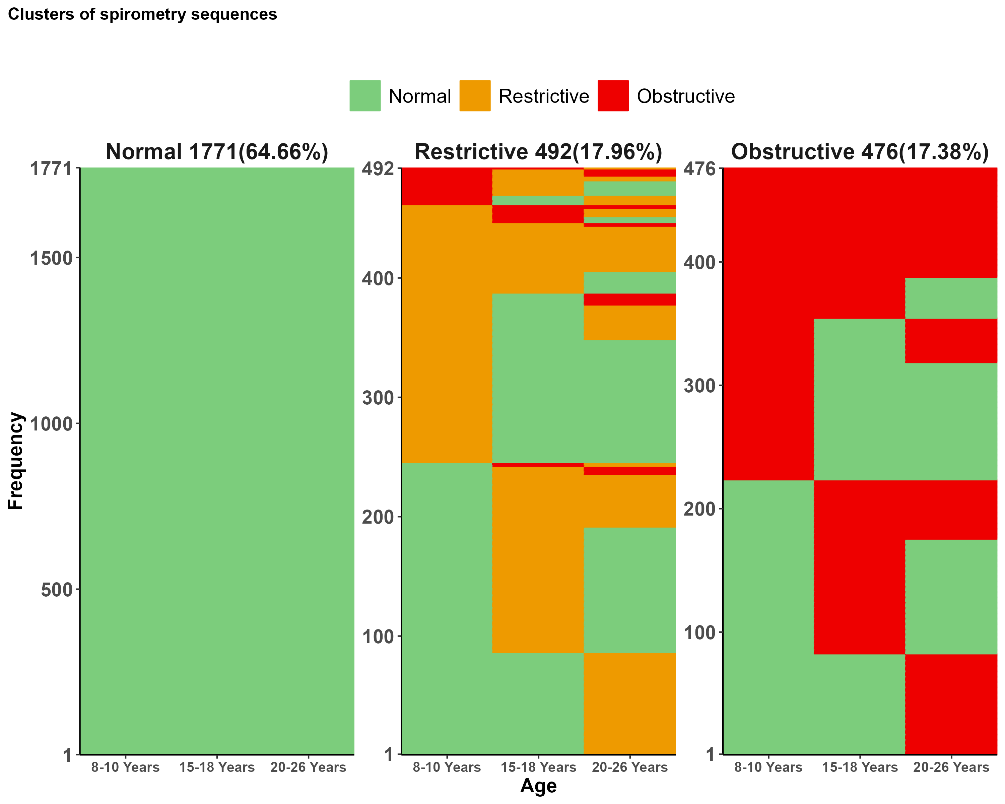
A)


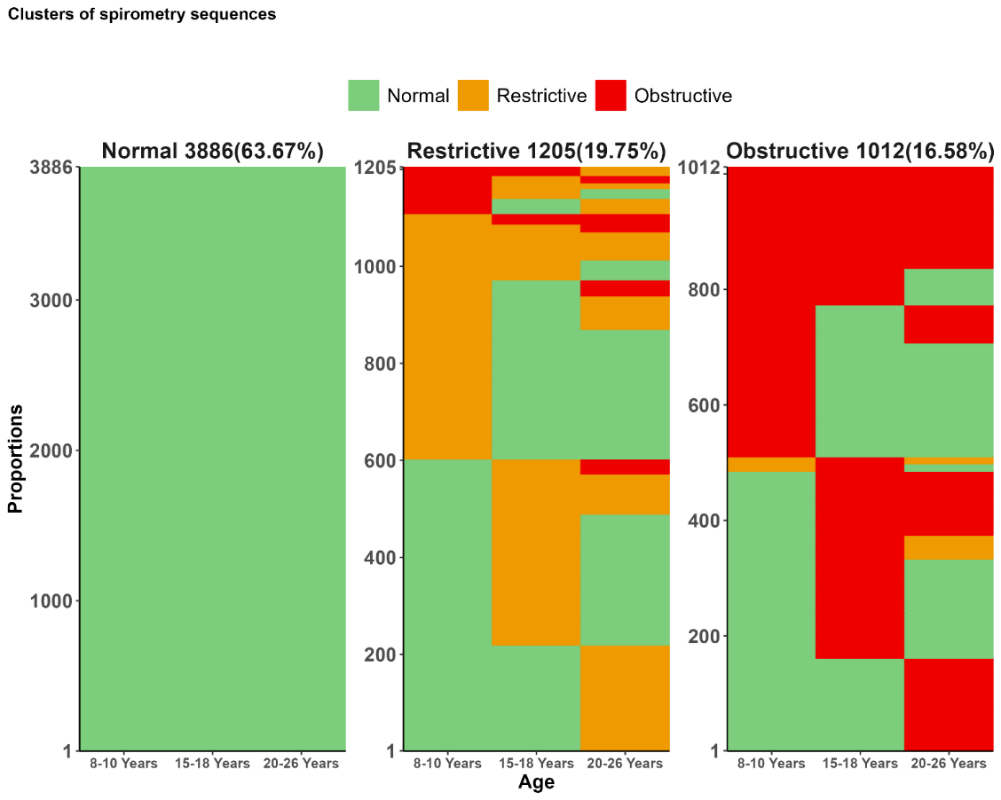
B)

**Figure S7:** Clusters of the spirometry sequences over time (individual cohort data). After classifying each participant as normal, restrictive, and obstructive at each time point, we clustered the individual participant's spirometry sequences. The grey colour represents missing values for the first or intermediate timepoints, whereas the white spaces represent loss to follow up.


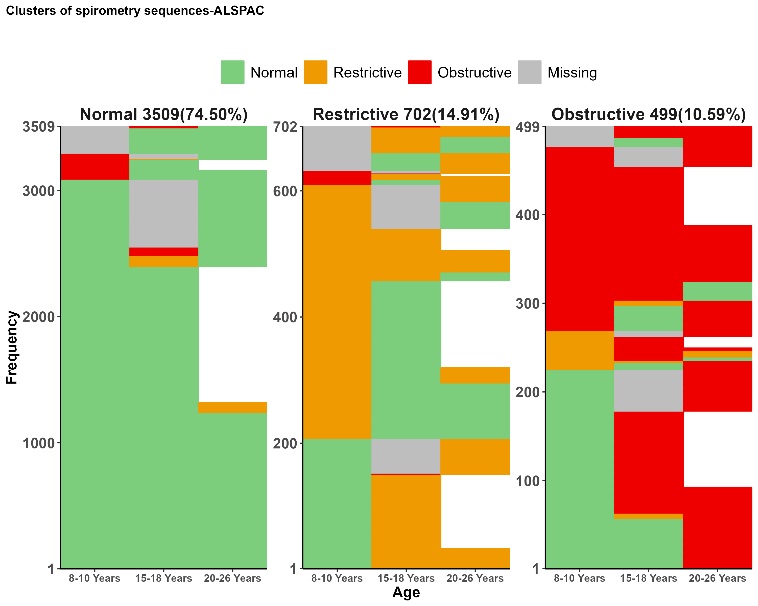

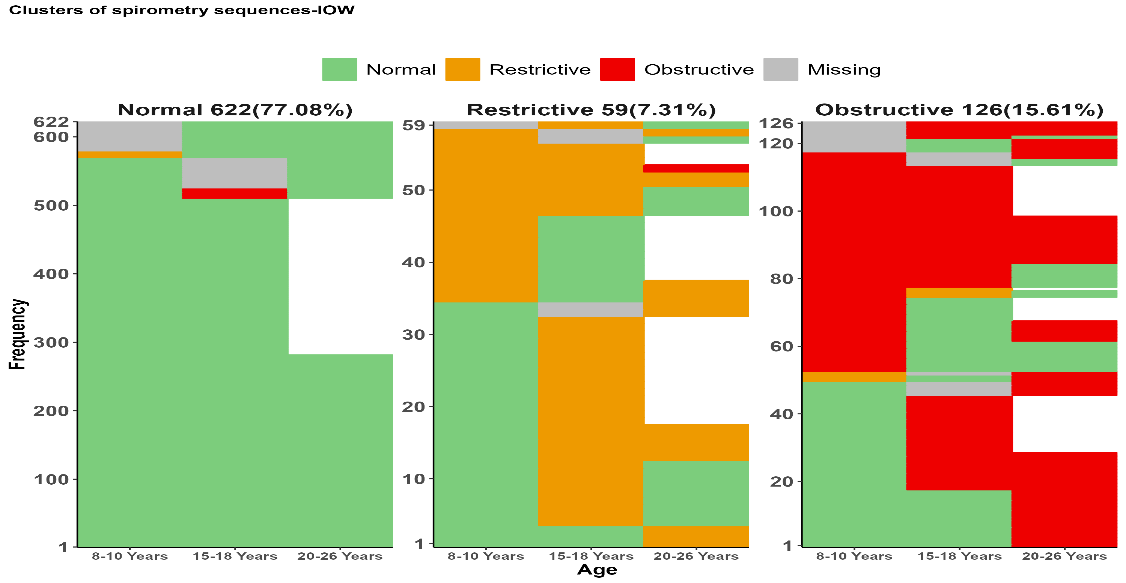

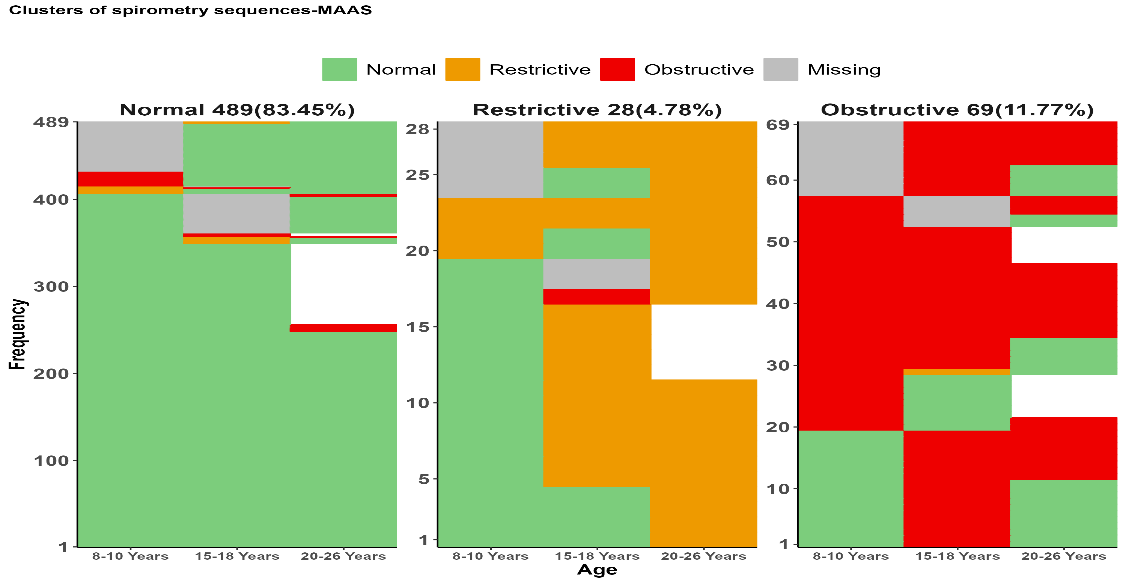


**Table S11:** Univariate risk factors of spirometry sequences clusters (Cluster-normal as a reference). Data are presented as odds ratio (OR) with a 95% confidence interval(CI). The odds ratios and 95% CI are estimated using a multinomial logistic regression model.

| **Risk factors** | **Univariate**  **OR (95% CI)**  **Cluster-Obstructive** | **P-value** | **Univariate**  **OR (95% CI)**  **Cluster-Restrictive** | **P-value** |
| --- | --- | --- | --- | --- |
| Male | 1.01 (0.94,1.09) | 0.683 | 1.08 (1.01,1.16) | **0.036** |
| Gestational age | 0.94 (0.917,0.98) | **0.003** | 0.97 (0.93,1.01) | 0.135 |
| Maternal age | 1.00 (0.98,1.01) | 0.730 | 1.00 (0.99,1.02) | 0.716 |
| Birth weight centiles (one unit=10 centiles increase) | 0.96 (0.94,0.99) | **0.009** | 0.97 (0.95,1.00) | 0.095 |
| Parental asthma | 1.04 (0.94,1.16) | 0.413 | 0.94 (0.84,1.07) | 0.349 |
| Maternal smoking during pregnancy | 1.22 (1.12,1.33) | **<.001** | 0.94 (0.84,1.05) | 0.268 |
| Breastfeeding for the first six months | 1.02 (0.92,1.13) | 0.714 | 1.01 (0.91,1.12) | 0.840 |
| Pet ownership in the first year of life^a^ | 1.04 (0.96,1.13) | 0.293 | 1.11 (1.02,1.21) | **0.015** |
| Early childhood wheeze^b^ | 1.49 (1.38,1.60) | **<.001** | 1.11 (1.03,1.20) | **0.009** |
| BMI z-score at first Lung function ^c^ | 1.09 (1.01,1.17) | **0.027** | 0.76 (0.70,0.83) | **<.001** |
| Current wheeze at first Lung function | 1.57 (1.45,1.71) | **<.001** | 1.00 (0.89,1.11) | 0.928 |
| Current asthma diagnosis at first Lung function | 1.59 (1.47,1.73) | **<.001** | 0.98 (0.88,1.09) | 0.704 |
| Current sensitization at first Lung function | 1.29 (1.18,1.41) | **<.001** | 1.00 (0.89,1.11) | 0.947 |
| Current Parental smoking at first Lung function | 1.11 (1.022,1.20) | **0.012** | 0.93 (0.85,1.01) | 0.090 |
| Underweightc^d^ | 0.87 (0.64,1.19) | 0.395 | 2.15 (1.68,2.77) | **<.001** |
| Overweight^d^ | 1.06 (0.88,1.27) | 0.527 | 0.65 (0.53,0.80) | **<.001** |
| Obese^d^ | 1.29 (0.99,1.68) | **0.062** | 0.81 (0.58,1.11) | 0.190 |
| 1. Pet inside the house or contact with pets most of the time in first year of life. 2. Current wheeze at age five or below years. 3. Based on the British 1990 Growth Reference 4. Defined using BMI z-score based on the British 1990 Growth Reference (underweight: z-score < -1, overweight: z-score > 1 and < 2, obese: z-score > 2). | | | | |

**Table S12:** Variance inflation factors for actual and imputed data.

| Risk factors | **VIF**  **Actual data** | **VIF**  **Imputed data** |
| --- | --- | --- |
| Male | 1.04668 | 1.04877 |
| Gestational age | 1.06514 | 1.06266 |
| Maternal age | 1.01686 | 1.01162 |
| Birth weight centiles | 1.06058 | 1.05416 |
| Parental asthma | 1.01812 | 1.00882 |
| Maternal smoking during pregnancy | 1.1263 | 1.09277 |
| Breastfeeding during the first six months | 1.03576 | 1.02962 |
| Pet ownership first year of life | 1.01818 | 1.0248 |
| Early childhood wheeze | 1.10521 | 1.11359 |
| BMI z-score at first LF | 3.16751 | 3.24305 |
| Current wheeze at first LF | 2.32696 | 1.93517 |
| Current asthma diagnosis at first LF | 2.38837 | 2.01217 |
| Current sensitization at first LF | 1.13702 | 1.15686 |
| Current Parental smoking at first LF | 1.0626 | 1.02626 |
| BMI categories at first LF | 3.15222 | 3.19639 |

**Figure S8:** Predicted probability of cluster membership by BMI z-score (pooled data). Points represent the predicted probability of each cluster membership for each participant based on his BMI at the time of first lung function. The probabilities are estimated from a mixed-effect multinomial logistic regression model with BMI z-score as a fixed effect covariate and cohort as a random effect.

**
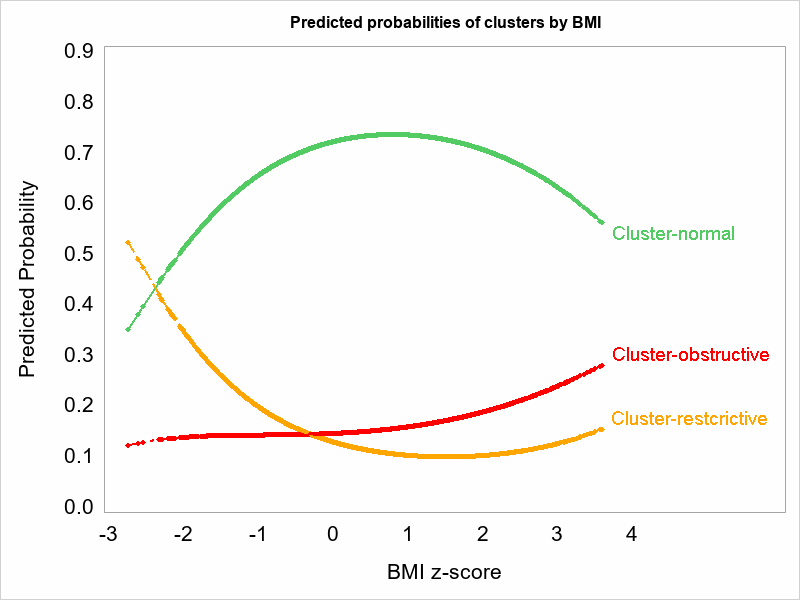
**

**Figure S9:** Predicted probability of cluster membership by BMI z-score and asthma diagnosis (pooled data). Points represent the predicted probability of each cluster membership for each participant based on his BMI and current asthma diagnosis at the time of first lung function. The probabilities are estimated from a mixed-effect multinomial logistic regression model with BMI z-score, BMI z-score(quadratic), current asthma diagnosis as fixed effect covariates, and cohort as a random effect.


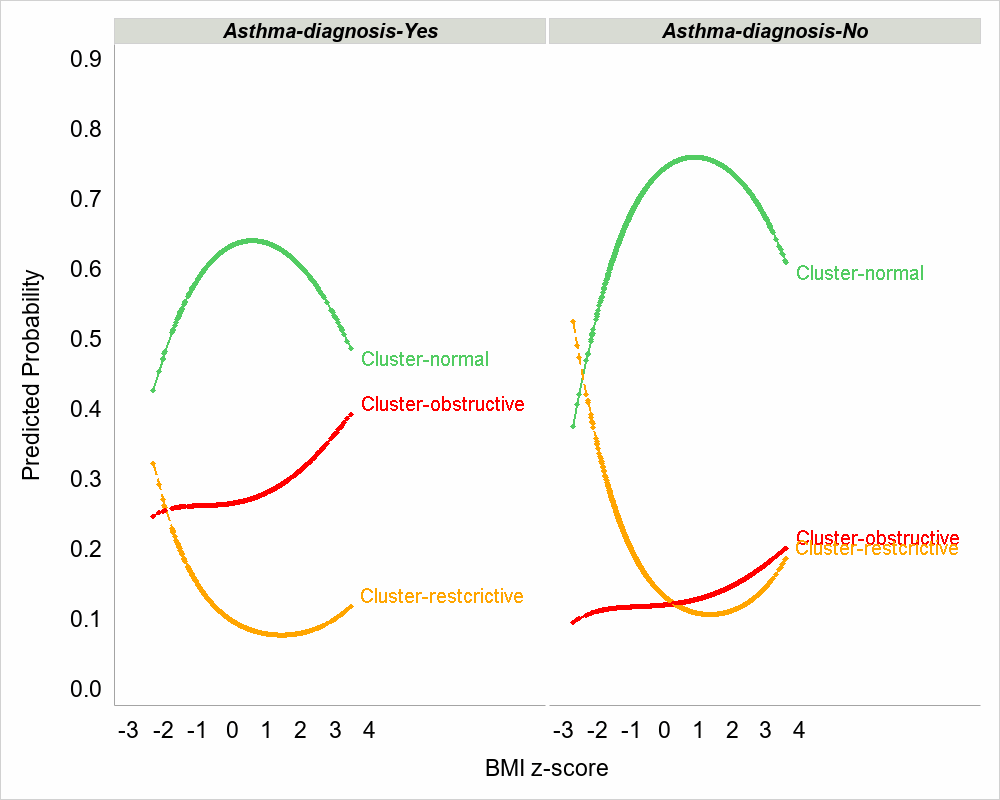


**Figure S10:** Predicted probabilities of clusters membership by BMI and wheeze phenotypes (NWZ: never; ETW: early-transient; INT: intermittent; LOW: late-onset; PEW: persistent).

Points represent the predicted probability of each cluster membership for each participant based on BMI category at the time of first lung function and PAM wheeze phenotype. The probabilities are estimated from a mixed-effect multinomial logistic regression model with BMI categories, wheeze phenotypes as fixed effect covariates, and cohort as a random effect. The model was adjusted for sex, gestational age, parental asthma, and maternal smoking during pregnancy. The Red dashed line shows the overall proportion of clusters.


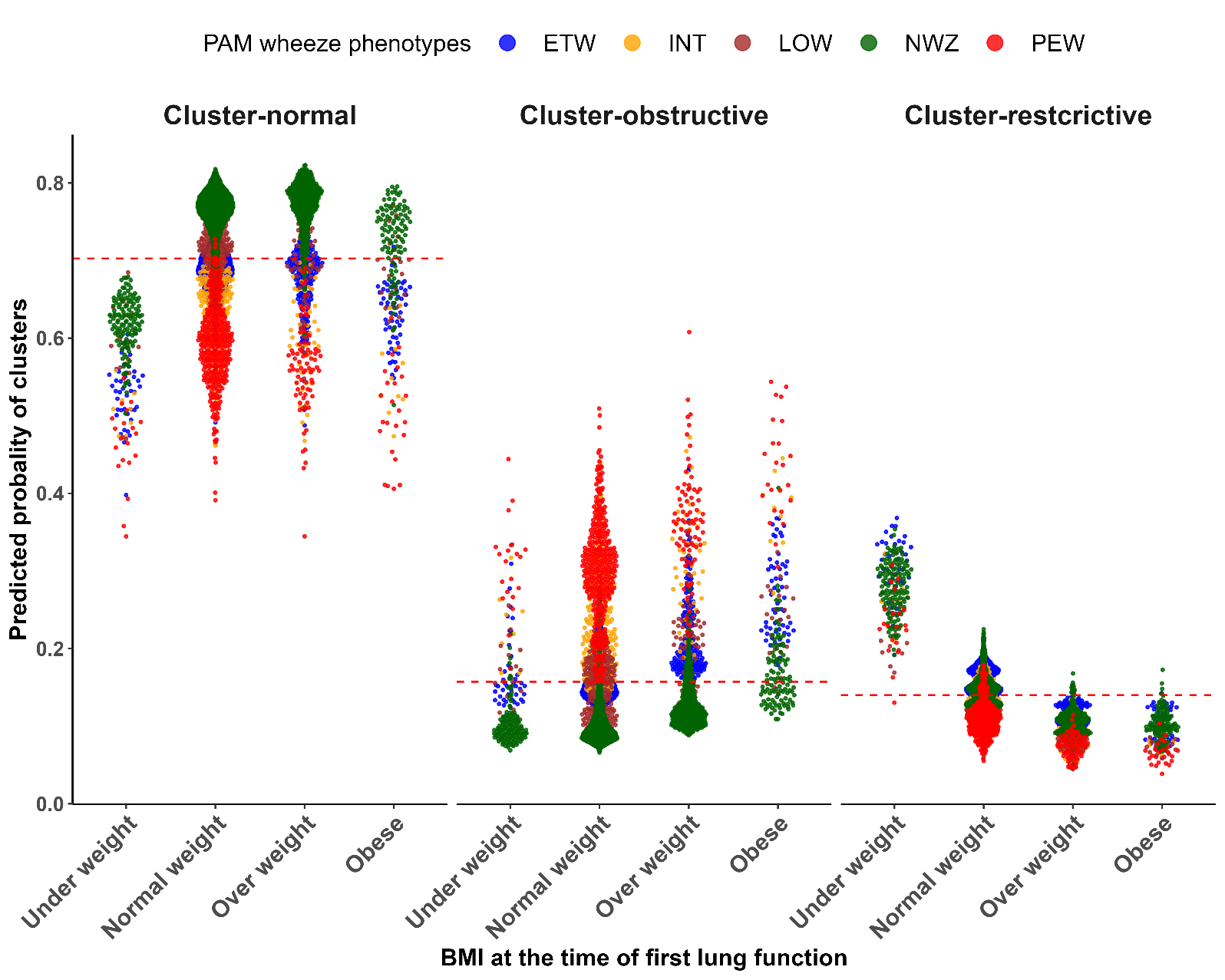


**REFERENCES**

1. Custovic A, Simpson BM, Murray CS, et al. The National Asthma Campaign Manchester Asthma and Allergy Study. *Pediatr Allergy Immunol* 2002; **13**(s15): 32-7.

2. Boyd A, Golding J, Macleod J, Lawlor DA, Fraser A, Henderson J, Molloy L, Ness A, Ring S, Davey Smith G. Cohort Profile: The 'Children of the 90s'--the index offspring of the Avon Longitudinal Study of Parents and Children. *Int J Epidemiol* 2013; **42**(1): 111-27.

3. Fraser A, Macdonald-Wallis C, Tilling K, et al. Cohort Profile: the Avon Longitudinal Study of Parents and Children: ALSPAC mothers cohort. *Int J Epidemiol* 2013; **42**(1): 97-110.

4. Northstone K, Lewcock M, Groom A, et al. The Avon Longitudinal Study of Parents and Children (ALSPAC): an update on the enrolled sample of index children in 2019. *Wellcome Open Res* 2019; **4**: 51.

5. Harris PA, Taylor R, Thielke R, Payne J, Gonzalez N, Conde JG. Research electronic data capture (REDCap)--a metadata-driven methodology and workflow process for providing translational research informatics support. *J Biomed Inform* 2009; **42**(2): 377-81.

6. Kurukulaaratchy RJ, Fenn M, Twiselton R, Matthews S, Arshad SH. The prevalence of asthma and wheezing illnesses amongst 10-year-old schoolchildren. *Respir Med* 2002; **96**(3): 163-9.

7. Kurukulaaratchy RJ, Fenn MH, Waterhouse LM, Matthews SM, Holgate ST, Arshad SH. Characterization of wheezing phenotypes in the first 10 years of life. *Clin Exp Allergy* 2003; **33**(5): 573-8.

8. Arshad SH, Holloway JW, Karmaus W, et al. Cohort Profile: The Isle Of Wight Whole Population Birth Cohort (IOWBC). *Int J Epidemiol* 2018; **47**(4): 1043-4i.

9. Miller MR, Hankinson J, Brusasco V, et al. Standardisation of spirometry. *Eur Respir J* 2005; **26**(2): 319-38.

10. Beydon N, Davis SD, Lombardi E, et al. An official American Thoracic Society/European Respiratory Society statement: pulmonary function testing in preschool children. *Am J Respir Crit Care Med* 2007; **175**(12): 1304-45.

11. Lodrup Carlsen KC, Roll S, Carlsen KH, et al. Does pet ownership in infancy lead to asthma or allergy at school age? Pooled analysis of individual participant data from 11 European birth cohorts. *PLoS One* 2012; **7**(8): e43214.

12. Roberts G, Peckitt C, Northstone K, et al. Relationship between aeroallergen and food allergen sensitization in childhood. *Clin Exp Allergy* 2005; **35**(7): 933-40.

13. Haider S, Granell R, Curtin J, et al. Modeling Wheezing Spells Identifies Phenotypes with Different Outcomes and Genetic Associates. *Am J Respir Crit Care Med* 2022; **205**(8): 883-93.

14. Studer M, Ritschard G. What matters in differences between life trajectories: a comparative review of sequence dissimilarity measures. *J R Stat Soc a Stat* 2016; **179**(2): 481-511.

15. Schafer JL. Analysis of incomplete multivariate data. London ; New York: Chapman & Hall; 1997.

16. Yuan Y. Multiple Imputation Using SAS Software. *J Stat Softw* 2011; **45**(6): 1-25.

17. Villar J, Ismail LC, Victora CG, et al. International standards for newborn weight, length, and head circumference by gestational age and sex: the Newborn Cross-Sectional Study of the INTERGROWTH-21st Project. *Lancet* 2014; **384**(9946): 857-68.

18. Vidmar SI, Cole TJ, Pan HQ. Standardizing anthropometric measures in children and adolescents with functions for egen: Update. *Stata J* 2013; **13**(2): 366-78.
